# Combining TIGIT blockade with IL‐15 stimulation is a promising immunotherapy strategy for lung adenocarcinoma

**DOI:** 10.1002/ctm2.1553

**Published:** 2024-01-27

**Authors:** Baohong Luo, Yu Sun, Qinru Zhan, Yuting Luo, Yu Chen, Tongze Fu, Tiantian Yang, Lijuan Ren, Zhongpeng Xie, Xiaohua Situ, Bixia Liu, Kejing Tang, Zunfu Ke

**Affiliations:** ^1^ Molecular Diagnosis and Gene Test Center The First Affiliated Hospital of Sun Yat‐sen University Guangzhou Guangdong China; ^2^ Department of Pathology The First Affiliated Hospital of Sun Yat‐sen University Guangzhou Guangdong China; ^3^ Department of Pathology Guangdong Provincial People's Hospital Guangdong Academy of Medical Sciences Guangzhou Guangdong China; ^4^ Division of Pulmonary and Critical Care Medicine The First Affiliated Hospital Sun Yat‐sen University Guangzhou Guangdong China

**Keywords:** IL‐15, immunotherapy, lung adenocarcinoma, TIGIT

## Abstract

**Background:**

T‐cell immunoglobulin and immunoreceptor tyrosine‐based inhibitory motif domain (TIGIT) is an immune checkpoint molecule that suppresses CD8^+^ T‐cell function in cancer. However, the expression profile and functional significance of TIGIT in the immune microenvironment of lung adenocarcinoma (LUAD) remain elusive. Interleukin (IL)‐15 has emerged as a promising candidate for enhancing CD8^+^ T‐cell mediated tumour eradication. Exploring therapeutic strategies that combine IL‐15 with TIGIT blockade in LUAD is warranted.

**Methods:**

We investigated the regulatory network involving coinhibitory TIGIT and CD96, as well as costimulatory CD226 in LUAD using clinical samples. The potential role of TIGIT in regulating the pathogenesis of LUAD was addressed through a murine model with transplanted tumours constructed in *Tigit* ^−/−^ mice. The therapeutic strategy that combines TIGIT blockade with IL‐15 stimulation was verified using a transplanted tumour murine model and a patient‐derived organoid (PDO) model.

**Results:**

The frequency of TIGIT^+^CD8^+^ T cells was significantly increased in LUAD. Increased TIGIT expression indicated poorer prognosis in LUAD patients. Furthermore, the effector function of TIGIT^+^CD8^+^ tumour‐infiltrating lymphocytes (TILs) was impaired in LUAD patients and TIGIT inhibited antitumour immune response of CD8^+^ TILs in tumour‐bearing mice. Mechanistically, IL‐15 enhanced the effector function of CD8^+^ TILs but stimulated the expression of TIGIT on CD8^+^ TILs concomitantly. The application of IL‐15 combined with TIGIT blockade showed additive effects in enhancing the cytotoxicity of CD8^+^ TILs and thus further increased the antitumour immune response in LUAD.

**Conclusions:**

Our findings identified TIGIT as a promising therapeutic target for LUAD. LUAD could benefit more from the combined therapy of IL‐15 stimulation and TIGIT blockade.

## Graphical abstract

1. The expression of coinhibitory TIGIT and CD96, as well as costimulatory CD226 on CD8^+^ T cells is dysregulated in LUAD, contributing to tumour invasion.
2. Mechanistically, IL‐15 enhances the effector function of CD8^+^ T cells but stimulates the upregulation of TIGIT concomitantly.
3. Our work highlights the potential clinical strategy of combining TIGIT blockade with IL‐15 stimulation for immunotherapy in LUAD.

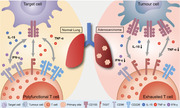

## 1 INTRODUCTION

Tumour‐infiltrating lymphocytes (TILs), particularly CD8^+^ TILs, are important in controlling tumour growth across various cancer types.^1^ Despite CD8^+^ TILs have a positive prognostic value in non‐small cell lung cancer (NSCLC),^2^ their abundant frequency fails to effectively promote tumour regression. Compelling evidence has suggested that the reactive CD8^+^ TILs from lung cancer patients become dysfunctional with impaired cytokine production and compromised proliferative capacity in the context of a broad spectrum of immunosuppressive signals occurring in the tumour microenvironment.^3^ The upregulation and persistent expression of inhibitory receptors, known as immune checkpoints, have emerged as the major markers of T‐cell dysfunction. Interfering with many of these receptors may unleash potent antitumour responses by harnessing the power of CD8^+^ TILs, thereby enhancing clinical outcomes for patients with NSCLC.^4^ Certainly, seminal research by Caron and colleagues defined and validated the correlation between increased expression of immune checkpoint molecules and clinical benefit from the blockade treatment in patients with advanced NSCLC.^5^ It is anticipated that the investigation of novel immune checkpoint molecules will provide further therapeutic options.

The immune checkpoint T‐cell immunoglobulin and immunoreceptor tyrosine‐based inhibitory motif domain (TIGIT) has recently been highlighted as an appealing target for new tumour immunotherapies. TIGIT is expressed in specific immune cells, including T cells, natural killer (NK) cells, and regulatory T cells (Tregs).^6^ TIGIT participates in an intricate regulatory network comprising multiple receptors and their corresponding ligands. The inhibitory TIGIT and CD96, along with the costimulatory CD226, compete for binding to the same ligand, CD155, which is similar to the CD28/CTLA‐4/CD80/CD86 pathway.^7^ Recently, accumulating evidence on the activity of this novel cosignalling pathway has highlighted TIGIT's capacity to suppress the T‐cell function.^8^ Blocking TIGIT may enhance T‐cell activation and restore exhaustion in preclinical murine models and cancer patients.^9^ Furthermore, several inhibitory receptors are frequently upregulated concurrently with the upregulation of TIGIT; as such, the simultaneous blockade of these coinhibitory receptor pathways reveals promising survival outcomes for NSCLC patients.^10^ These findings support the development of novel combinatorial immunotherapy strategies involving TIGIT blockade.

Although previous studies have reported a high expression of TIGIT in lung adenocarcinoma (LUAD),^11^, ^12^ the role of TIGIT in the immune response of CD8^+^ T cells and their functional exhaustion remains elusive; in particular, the molecular and functional relationship between TIGIT/CD96/CD226 in the immune microenvironment of LUAD merits further research. In this study, we present an evidence‐based discussion of efforts aimed at comprehending and harnessing TIGIT as an immunotherapy target for LUAD mediated by CD8^+^ T cells. Moreover, we determined that combining TIGIT blockade with interleukin (IL)‐15 stimulation could promote CD8^+^ T‐cell cytotoxicity, which offers a promising clinical strategy in the treatment of LUAD.

## 2 RESULTS

### 2.1 The expression of T‐cell immunoglobulin and immunoreceptor tyrosine‐based inhibitory motif domain is upregulated on CD8^+^ T cells in patients with lung adenocarcinoma

T cells and NK cells, major effector cells in regulating the immune response against tumours,^13^ were detected in LUAD tissues by immunohistochemistry. Intratumoural tissues of LUAD patients exhibited significantly higher proportions of CD4^+^ T cells and CD8^+^ T cells than peritumoural tissues; however, the proportions of CD56^+^ NK cells and CD4^+^CD25^+^Foxp3^+^ Tregs were very low, with no significant difference between intratumoural and peritumoural tissues (Figure S1). Therefore, T cells from various sources, including blood and different types of lung tissues, were subsequently analysed through flow cytometry (Figure S2). The cumulative percentages of peripheral CD4^+^ T cells and CD8^+^ T cells showed no significant difference between healthy controls (HCs) and patients with LUAD (Figures S3A and 1A). However, the cumulative percentages of CD4^+^ TILs and CD8^+^ TILs were found to be significantly higher in intratumoural tissues than those observed in paired peritumoural tissues of each LUAD patient (Figures S3A and 1A), suggesting a crucial role of T cells in LUAD tumourigenesis.

**FIGURE 1:**
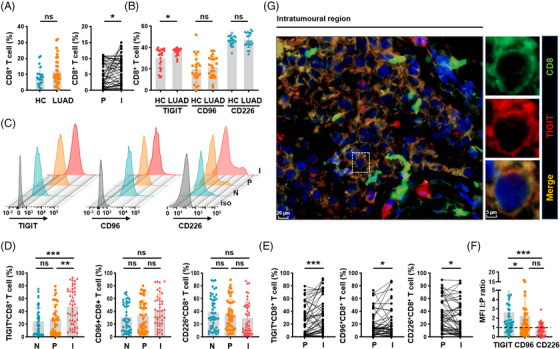
TIGIT expression is increased on both peripheral and intratumoural CD8^+^ T cells in patients with LUAD. (A) Cumulative percentage of peripheral CD8^+^ T cells in HCs and patients with LUAD (left) and in paired peritumoural and intratumoural tissue of each LUAD patient (right). (B) Cumulative percentage of TIGIT, CD96 or CD226 expression on peripheral CD8^+^ T cells. (C) Representative flow cytometry histogram showing TIGIT, CD96 or CD226 expression on CD3^+^CD8^+^ T cells in normal lung, peritumoural and intratumoural lung tissues. (D) Cumulative percentage of TIGIT^+^CD8^+^, CD96^+^CD8^+^ or CD226^+^CD8^+^ T cells in normal lung, peritumoural and intratumoural lung tissues. (E) Cumulative percentage of TIGIT^+^CD8^+^, CD96^+^CD8^+^ or CD226^+^CD8^+^ T cells in paired peritumoural and intratumoural tissue of each LUAD patient. (F) The relative MFI fold‐change of TIGIT, CD96 or CD226 on intratumoural CD8^+^ T cells compared to that on paired peritumoural CD8^+^ T cells. (G) Immunofluorescence image showing the colocalization of CD8 and TIGIT. Results are presented as the means ± SD of 22–50 independent individuals. *P < .05, **P < .01, ***P < .001, ns, no significant difference; Welch's *t*‐test (A, left), two‐tailed Student's *t‐*test (B), Kruskal‒Wallis ANOVA followed by Dunn's multiple comparisons test (D,F) and Wilcoxon matched‐pairs signed‐rank test (A, right, E) were used. HC, healthy control; P, peritumoural tissue; I, intratumoural tissue; N, normal lung tissue; Iso, isotype; MFI, mean fluorescence intensity; TIGIT, T‐cell immunoglobulin and immunoreceptor tyrosine‐based inhibitory motif domain; LUAD, lung adenocarcinoma; SD, standard deviation.

Next, the Kruskal‒Wallis nonparametric test and multiple comparisons analysis were used to evaluate the expression levels of TIGIT, CD96 and CD226 on CD8^+^ T cells. In contrast to HCs, LUAD patients had a significant increase in circulating TIGIT^+^CD8^+^ T cells (Figure 1B,C). Furthermore, the percentage of TIGIT^+^CD8^+^ T cells was notably higher in intratumoural tissues compared to the peritumoural and normal tissues, whereas the percentage of CD96^+^ and CD226^+^ CD8^+^ T cells remained relatively stable across different regions (Figure 1D). The disparities were further accentuated when comparing the paired intratumoural and peritumoural tissue of each individual patient (Figure 1E). The analysis of mean fluorescence intensity (MFI) confirmed that intratumoural CD8^+^ T cells expressed higher levels of TIGIT than peritumoural CD8^+^ T cells (intratumour: peritumour ratio > 1) (Figure 1F). In addition, the immunofluorescent staining imaging of TIGIT with CD8 revealed the coexpression and spatial overlap of TIGIT and CD8 on the cell membrane within the tumour nest (Figure 1G). We also assessed the expression levels of TIGIT, CD96 and CD226 on CD4^+^ T cells. Unlike CD8^+^ T cells, the percentage and MFI of TIGIT^+^, CD96^+^ or CD226^+^ CD4^+^ T cells showed no significant difference among normal lung, intratumoural and peritumoural tissues of LUAD patients (Figure S3B,D), although a significant difference was observed in the percentage of CD96^+^CD4^+^ T cells when comparing the paired intratumoural and peritumoural tissue (Figure S3C). Collectively, these findings suggest that TIGIT expression is increased specifically on CD8^+^ T cells, rather than CD4^+^ T cells, in patients with LUAD.

### 2.2 The distribution of T‐cell immunoglobulin and immunoreceptor tyrosine‐based inhibitory motif domain, CD96 and CD226 on CD8^+^ tumour‐infiltrating lymphocytes is imbalanced in patients with lung adenocarcinoma

**FIGURE 2:**
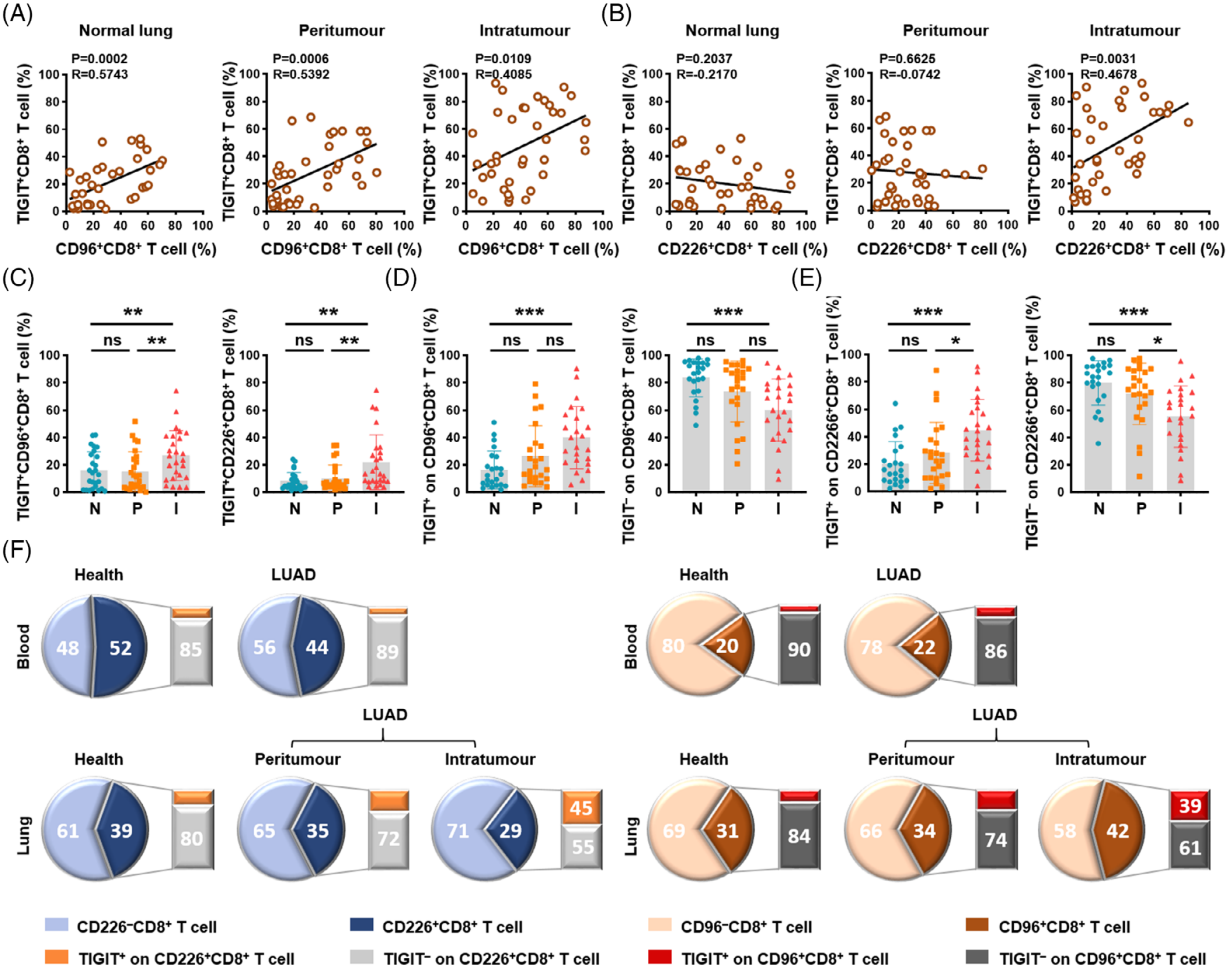
The balance of TIGIT, CD96 and CD226 expression on CD8^+^ T cells is disrupted in LUAD patients. (A,B) Correlation analysis between the percentage of TIGIT^+^CD8^+^ T cells and CD96^+^CD8^+^ T cells (A) or CD226^+^CD8^+^ T cells (B) in normal lung, peritumoural and intratumoural tissues of LUAD patients. (C) Cumulative percentage of TIGIT^+^CD96^+^CD8^+^ T cells and TIGIT^+^CD226^+^CD8^+^ T cells in normal, peritumoural and intratumoural lung tissues. (D,E) Percentage of TIGIT^+^ or TIGIT^–^ on CD96^+^CD8^+^ T cells (D) and percentage of TIGIT^+^ or TIGIT^–^ on CD226^+^CD8^+^ T cells (E) from normal, peritumoural and intratumoural lung tissues. (F, left) Percentage of CD226^+^CD8^+^ T cells in peripheral CD8^+^ T cells (top) or in tissue‐resident CD8^+^ T cells of normal, peritumoural and intratumoural lung tissues (bottom). The orange block shows the proportion of TIGIT expression within the total population of CD226^+^CD8^+^ T cells. (Right) Percentage of CD96^+^CD8^+^ T cells in peripheral CD8^+^ T cells (top) or in tissue‐resident CD8^+^ T cells of normal, peritumoural and intratumoural lung tissues (bottom). The red block shows the proportion of TIGIT expression within the total population of CD96^+^CD8^+^ T cells. Results are presented as the means ± SD of 24 independent individuals. *P < .05, **P < .01, ***P < .001, ns, no significant difference; simple linear regression (A,B) and Kruskal‒Wallis ANOVA followed by Dunn's multiple comparisons test (C–E) were used. N, normal lung tissue; P, peritumoural tissue; I, intratumoural tissue; TIGIT, T‐cell immunoglobulin and immunoreceptor tyrosine‐based inhibitory motif domain; LUAD, lung adenocarcinoma; SD, standard deviation.

The coinhibitory TIGIT and CD96, along with the costimulatory CD226, form an axis that transmits activation or inhibitory signals depending on the relative abundance of receptors available.^14^ To explore the potential impact of the tumour microenvironment on immune homeostasis between TIGIT, CD96 and CD226 in LUAD, we performed correlation analyses among these three receptors. In normal lung, peritumoural and intratumoural tissues, the percentage of TIGIT^+^CD8^+^ T cells positively correlated with the percentage of CD96^+^CD8^+^ T cells (Figure 2A). In particular, the positive association between TIGIT^+^CD8^+^ T cells and CD96^+^CD8^+^ T cells became gradually weakened from normal lung tissues (*R* = .5743) to peritumour specimens (*R* = .5392) and finally to intratumour specimens (*R* = .4085) (Figure 2A). In contrast, the percentage of TIGIT^+^CD8^+^ T cells was negatively associated with the percentage of CD226^+^CD8^+^ T cells in normal lungs (*R* = −.2170), although the correlation was not statistically significant (Figure 2B). However, the negative correlations became less obvious in peritumoural specimens (*R* = −.0742) and were reversed in intratumoural specimens (*R* = .4678) (Figure 2B).

The proportions of TIGIT^+^CD96^+^ and TIGIT^+^CD226^+^ subsets within CD8^+^ T cells were analysed to confirm the dysregulation of TIGIT, CD96 and CD226 expression. The percentages of TIGIT^+^CD96^+^ and TIGIT^+^CD226^+^ populations were significantly elevated in intratumoural tissues compared to peritumoural and normal lung tissues (Figure 2C). Meanwhile, the proportion of CD96^+^CD8^+^ T cells was assessed based on their expression of TIGIT; the percentage of TIGIT^+^ cells on CD96^+^CD8^+^ T cells was significantly higher in intratumoural tissues than in peritumoural and normal lung tissues, whereas the percentage of TIGIT^–^ cells on CD96^+^CD8^+^ T cells was significantly reduced (Figure 2D). These findings were consistently observed in CD226^+^CD8^+^ T cells (Figure 2E), indicating that the transition of CD96^+^CD8^+^ and CD226^+^ CD8^+^ T cells from the TIGIT^–^ to TIGIT^+^ phenotype may occur once they reach the cancer nest. Moreover, in healthy blood and lung specimens, the TIGIT^+^CD226^+^ and TIGIT^+^CD96^+^ subsets accounted for only minor fractions of CD226^+^ and CD96^+^ CD8^+^ T cells, respectively (Figure 2F). However, these subsets were significantly expanded in intratumoural specimens (Figure 2F). Together, these findings suggest that TIGIT expression is upregulated on both CD96^+^CD8^+^ T cells and CD226^+^CD8^+^ T cells within the tumour microenvironment of LUAD.

### 2.3 T‐cell immunoglobulin and immunoreceptor tyrosine‐based inhibitory motif domain dysregulation is an indicator of unfavorable disease conditions and poor prognosis in patients with lung adenocarcinoma

**FIGURE 3:**
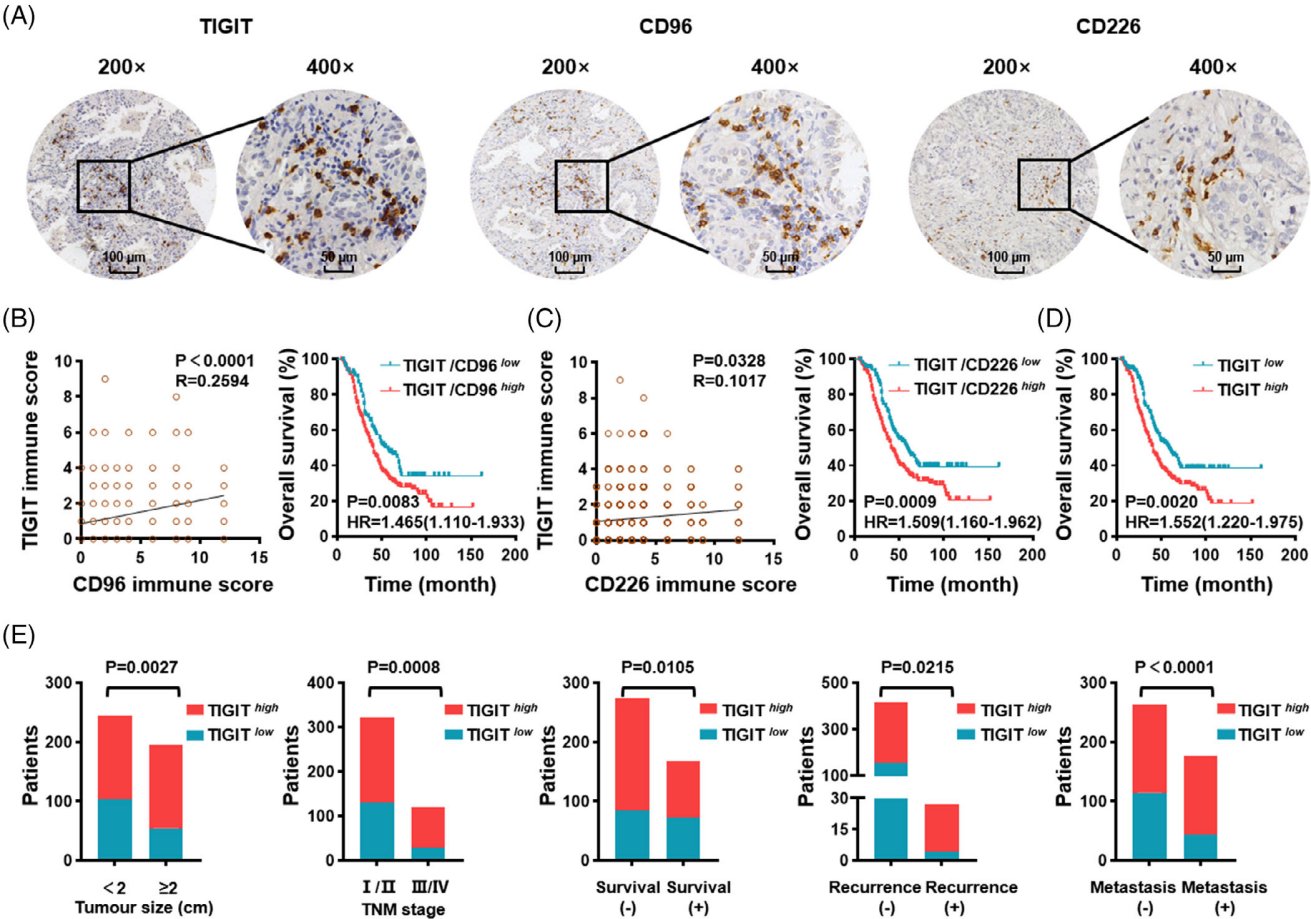
Dysregulation of TIGIT correlates with unfavorable clinical prognosis in LUAD patients. (A) Representative microscopy images showing TIGIT, CD96 and CD226 immunostaining in lymphocytes infiltrated in LUAD. Original magnifications: ×200 (right) and ×400 (left). (B, left) Correlation analysis between TIGIT immune scores and CD96 immune scores (*N* = 441). (Right) Kaplan‒Meier survival curves for overall survival according to high and low TIGIT/CD96 ratios. (C, left) Correlation analysis between TIGIT immune scores and CD226 immune scores (*N* = 441). (Right) Kaplan‒Meier survival curves for overall survival according to high and low TIGIT/CD226 ratios. (D) Kaplan‒Meier survival curves for overall survival according to the level of intratumoural TIGIT expression. (E) Comparison of tumour sizes, TNM stages, survival durations, recurrence or metastasis statuses between patients with higher TIGIT scores and those with lower scores. Simple linear regression (B,C), log‐rank test (B–D) and chi‐square test (E) were used. LUAD, lung adenocarcinoma; TIGIT, T‐cell immunoglobulin and immunoreceptor tyrosine‐based inhibitory motif domain; TNM, umour‐node‐metastasis.

Although a previous study elucidated the prognostic significance of the combined evaluation of TIGIT and CD155 in LUAD,^12^ it remains unclear whether the dysregulated relationship between TIGIT, CD96 and CD226 expression influences clinical outcomes. To address this, immunohistochemical analysis was performed by two pathologists (Figure 3A) to semiquantify TIGIT, CD96, and CD226 expression in 441 LUAD samples using a minimum *P*‐value threshold approach.^15^ The immunoscore of TIGIT was positively correlated with the immunoscore of CD96 (*R* = .2594) and CD226 (*R* = .1017; Figure 3B,C, left), suggesting the consistency of the findings presented in Figure 2A,B. Based on their TIGIT and CD96 scores, LUAD patients were then divided into groups with high and low TIGIT/CD96 ratios. The overall survival was much shorter in patients with a high TIGIT/CD96 ratio than those with a low ratio (*P* = .0083; Figure 3B, right). Similar results were found in the groups with high or low TIGIT/CD226 ratios (*P* = .0009; Figure 3C, right), indicating that an increased TIGIT ratio may potentially impede the survival of LUAD patients. The immunoscores of TIGIT and CD155 also showed a positive association, and as expected, patients with an increased density of TIGIT^+^CD155^+^ had shorter overall survival (Figure S4C–H). Patients were further categorised based on their individual TIGIT, CD96 and CD226 scores. Patients with higher TIGIT scores exhibited a shorter overall survival time than those with lower scores (*P* = .0020; Figure 3D, Tables S1 and S2). However, no significant prognostic value of CD96 and CD226 in predicting overall survival time was observed (Figure S4A,B, Tables S1 and S2). Chi‐square analysis of the clinical information of LUAD patients revealed a significant association between higher TIGIT score and bigger tumour size, advanced tumour‐node‐metastasis (TNM) stage, worse survival status, as well as an increased risk of recurrence and metastasis compared to patients with lower scores (Figure 3E). Altogether, these data validate the dysregulation of TIGIT as an indicator of prognosis in patients with LUAD.

### 2.4 TIGIT^+^CD8^+^ tumour‐infiltrating lymphocytes in lung adenocarcinoma are functionally exhausted

**FIGURE 4:**
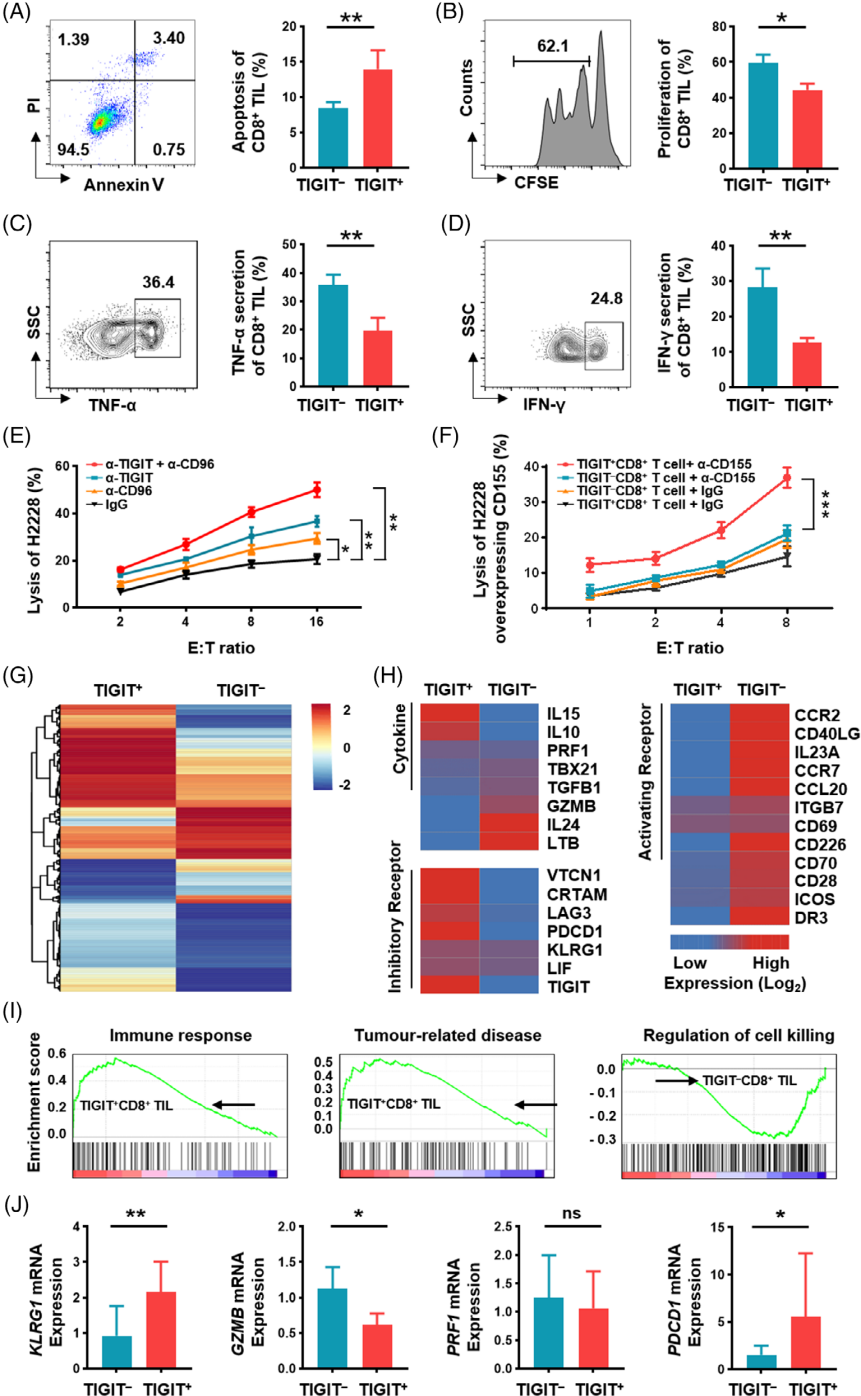
TIGIT exhibits an inhibitory effect on CD8^+^ T cells. (A–D) Comparison of the apoptotic percentage (A), proliferation rate (B), TNF‐α secreting percentage (C), and IFN‐γ secreting percentage (D) between primary TIGIT^+^CD8^+^ TILs and TIGIT^–^CD8^+^ TILs in LUAD. Representative flow cytometry on TIGIT^–^CD8^+^ TILs are shown. (E) Lysis of H2228 cells in the presence of anti‐TIGIT (green), anti‐CD96 (yellow), a combination of both antibodies (red), or control antibody (black). (F) Lysis of H2228 cells with CD155 overexpression by primary TIGIT^+^CD8^+^ T cells with anti‐CD155 (red), TIGIT^–^CD8^+^ T cells with anti‐CD155 (green), TIGIT^+^CD8^+^ T cells with control antibody (yellow) or TIGIT^–^CD8^+^ T cells with control antibody (dark). (G) Heatmap showing mRNA transcripts up‐ or downregulated in the TIGIT^+^CD8^+^ and TIGIT^–^CD8^+^ TIL subsets. *Z* score on a log2 scale: −2 to 2. (H) Heatmaps showing representative transcripts of cytokines, inhibitory receptors, or activating receptors in TIGIT^+^CD8^+^ and TIGIT^–^CD8^+^ TILs. *Z* score on a log2 scale: −.5 to.5. (I) GSEA plot showing gene signatures associated with the regulation of immune response (left), tumour‐related disease (middle), or regulation of cell killing (right) in TIGIT^+^CD8^+^ TILs relative to TIGIT^–^CD8^+^ TILs. (J) Comparison of KLRG1, GZMB, PRF1 and PDCD1 mRNA levels between TIGIT^+^CD8^+^ and TIGIT^–^CD8^+^ TILs by real‐time PCR analysis. Results are presented as the means ± SD of at least three independent experiments. *P < .05, **P < .01, ***P < .001. ns, no significant difference; two‐tailed Student's *t*‐test (A, B, C, D, J) and two‐way ANOVA with Tukey's post hoc test (E, F) were used. GSEA, gene set enrichment analysis; IFN, interferon; LUAD, lung adenocarcinoma; PCR, polymerase chain reaction; SD, standard deviation; TIGIT, T‐cell immunoglobulin and immunoreceptor tyrosine‐based inhibitory motif domain; TNF, tumour necrosis factor; TIL, tumour‐infiltrating lymphocyte.

To investigate the influence of TIGIT expression on the CD8^+^ T‐cell activity, the viability of TIGIT^+^CD8^+^ TILs and TIGIT^–^CD8^+^ TILs was assessed in patients with LUAD. The apoptotic rate of TIGIT^+^CD8^+^ TILs was significantly increased, while their proliferative capacity was significantly reduced compared to that of TIGIT^–^CD8^+^ TILs (Figure 4A,B). Moreover, the ability of TIGIT^+^CD8^+^ TILs to produce interferon (IFN)‐γ and tumour necrosis factor (TNF)‐α was significantly impaired (Figure 4C,D), indicating that TIGIT may hinder the cytotoxic function of CD8^+^ T cells. To further clarify the inhibitory role of TIGIT on CD8^+^ T‐cell‐mediated cytotoxicity, we performed a blockade assay using an anti‐TIGIT antibody. The blockade of TIGIT resulted in a significant increase in H2228 cell lysis; additionally, the simultaneous blockade of TIGIT and CD96 further augmented the cytotoxic activity of CD8^+^ T cells (Figure 4E). TIGIT acts as an inhibitory receptor by binding to its ligand CD155, thereby transmitting of negative signals to effector cells.^16^ To investigate the effects of blocking the TIGIT–CD155 interaction, H2228 cells stably overexpressing CD155 were generated as target cells. Subsequently, TIGIT^+^CD8^+^ and TIGIT^–^CD8^+^ TILs were isolated separately as effector cells. The presence of anti‐CD155 and TIGIT^+^CD8^+^ T cells resulted in a significantly enhanced cytotoxicity against H2228 cells overexpressing CD155 (Figure 4F). Altogether, these data indicate that inhibition of TIGIT–CD155 interaction can greatly enhance the cytotoxicity of TIGIT^+^CD8^+^ T cells against LUAD.

Next, we isolated and purified TIGIT^+^CD8^+^ TILs and TIGIT^–^CD8^+^ TILs from LUAD tissues through magnetic bead separation and flow cytometry sorting. Global gene expression profiling analysis showed that there were greater than two‐fold differences between TIGIT^+^CD8^+^ TILs and TIGIT^–^CD8^+^ TILs in 673 expressed genes (Figures 4G and S5). Quantitative analysis revealed an upregulation of inhibitory molecules, including TIGIT, PD‐1, and Lag‐3, and a downregulation of activation‐related molecules, such as CD28 and CD226, in TIGIT^+^CD8^+^ TILs (Figure 4H). Importantly, TIGIT^+^CD8^+^ TILs exhibited higher expression levels of IL‐10 and IL‐15 and lower expression levels of T‐bet, granzyme B and transforming growth factor (TGF)‐β (Figure 4H). Furthermore, through gene set enrichment analysis (GSEA), we found that the upregulated genes were enriched in gene sets associated with immune response regulation and tumourigenesis (Figure 4I). Conversely, the downregulated genes were enriched for signatures related to cell killing (Figure 4I). The upregulation of PD‐1 and KLRG1, along with the downregulation of granzyme B in TIGIT^+^CD8^+^ TILs, was confirmed by quantitative polymerase chain reaction (PCR) (Figure 4J). Collectively, these findings suggest that TIGIT^+^CD8^+^ TILs exhibit functional exhaustion compared to TIGIT^–^CD8^+^ TILs in LUAD.

### 2.5 T‐cell immunoglobulin and immunoreceptor tyrosine‐based inhibitory motif domain inhibits the antitumour immune response of CD8^+^ tumour‐infiltrating lymphocytes in vivo

**FIGURE 5:**
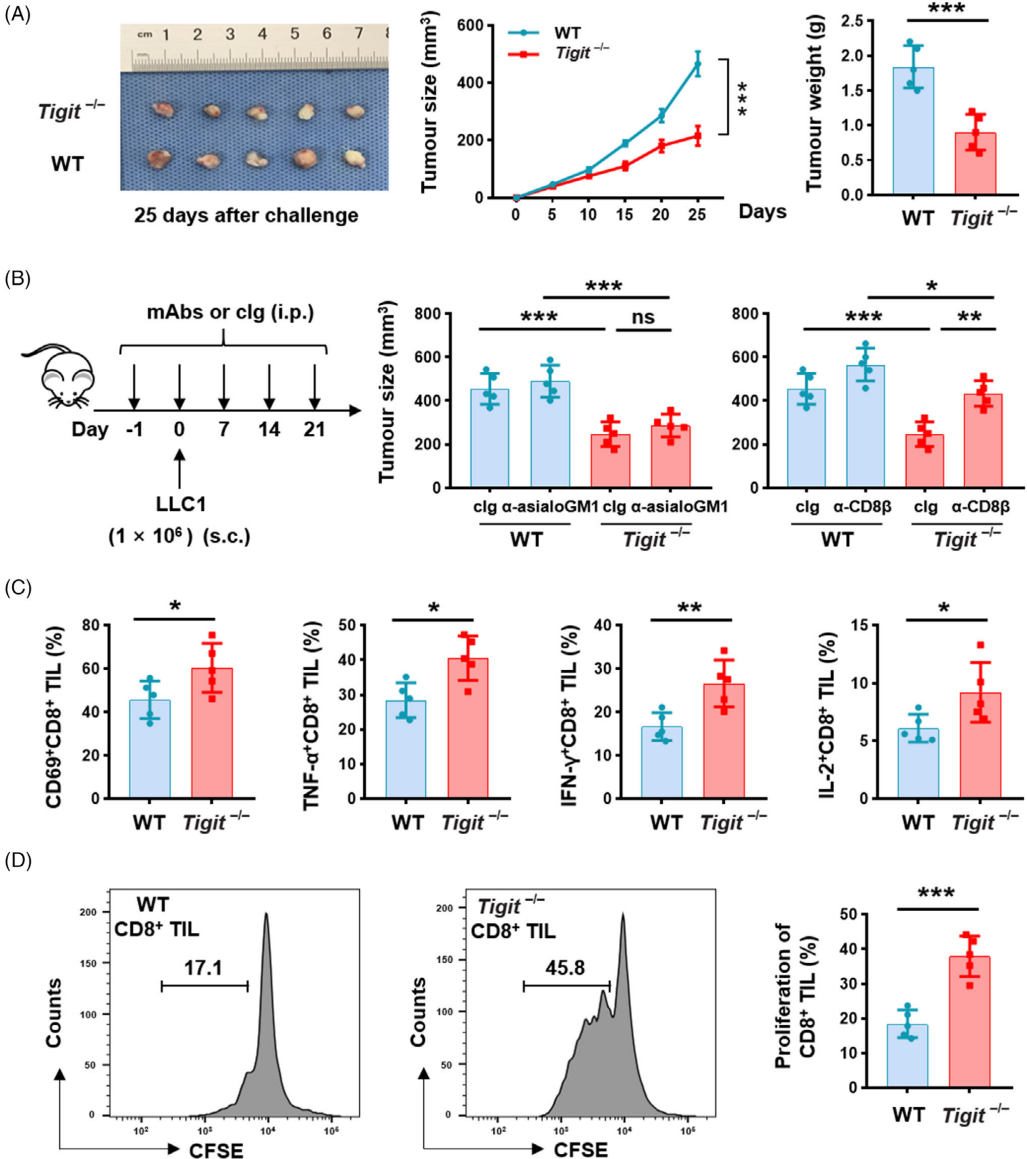
TIGIT inhibits the antitumour immunity of CD8^+^ T cells. (A) Quantitation of tumour volume and tumour weight of *Tigit* ^−/−^ and wild‐type (WT) mice inoculated subcutaneously (s.c.) with 1 × 10^6^ LLC1 cells (five mice in each group). (B) Experiment scheme: *Tigit* ^−/−^ and WT mice were inoculated s.c. with 1 × 10^6^ LLC1 cells on Day 0 and intraperitoneally (i.p.) injected with anti‐asialoGM1, or anti‐CD8β, or their control immunoglobulin (cIg) on Days −1, 0, 7, 14, and 21. Comparison of tumour size between *Tigit* ^−/−^mice treated with depletion antibody and cIg. (C) TILs were isolated from *Tigit* ^−/−^ and C57BL/6 WT mice, followed by stimulation with anti‐CD3/CD28 microbeads for a duration of 6 h. The expression levels of CD69, TNF‐α, IFN‐γ and IL‐2 within the CD8^+^ TIL population are shown. (D) Representative flow cytometry histogram showing CFSE dilution in CD8^+^ TILs. The proliferation percentage of CD8^+^ TILs isolated from *Tigit^−/−^* and C57BL/6 WT mice is shown. Results are presented as the means ± SD and data shown are representative of three independent experiments. *P < .05, **P < .01, ***P < .001, ns, no significant difference; two‐tailed Student's *t*‐test (A, C, D) and two‐way ANOVA (A), and two‐way ANOVA with Tukey's post hoc test (B) were used. CFSE, carboxyfluorescein diacetate succinimidyl ester; IFN, interferon; IL, interleukin; SD, standard deviation; TIGIT, T‐cell immunoglobulin and immunoreceptor tyrosine‐based inhibitory motif domain; TIL, tumour‐infiltratinglymphocyte; TNF, tumour necrosis factor.

Our data have demonstrated that TIGIT^+^CD8^+^ T cells in LUAD are functionally exhausted (Figure 4); however, the potential role of TIGIT in regulating lung cancer pathogenesis remains elusive. To address this, a transplanted tumour murine model was constructed using LLC1 cell lines in germline TIGIT‐deficient (*Tigit* ^−/−^) mice and wild‐type (WT) littermates. TIGIT depletion significantly delayed the growth of LLC1 lung carcinoma compared to WT controls (Figure 5A). As TIGIT is expressed mainly on T cells, NK cells and Tregs, we aimed to determine whether the effector functions of TIGIT deficiency were dependent on either of these cell populations. The depletion antibodies specific for CD8, CD4, asialo‐GM1, or CD25 were administrated to effectively eliminate the majority of CD8^+^ T cells, CD4^+^ T cells, NK cells or Tregs (Figure S6A). The antitumour effects in *Tigit* ^−/−^ mice were abolished when depletion of CD8^+^ T cells, rather than NK cells, CD4^+^ T cells, or Tregs (Figures 5B and S6B,C), indicating that CD8^+^ T cells are essential for inhibiting tumour growth when TIGIT is depleted. Furthermore, the percentage of CD8^+^ TILs producing TNF‐α, IFN‐γ, or IL‐2 was found to be significantly higher in *Tigit* ^−/−^ mice than in WT mice (Figures 5C and S7), suggesting that TIGIT depletion is indeed associated with enhanced functionality in CD8^+^ TILs. Then, the proliferative potential of CD8^+^ TILs was assessed through anti‐CD3 stimulation. CD8^+^ TILs derived from *Tigit* ^−/−^ mice exhibited an augmented proliferative response compared to those derived from WT mice (Figure 5D). Altogether, these findings suggest that TIGIT restrains the effectiveness of CD8^+^ T cells in eliminating LUAD.

### 2.6 The intratumoural interleukin‐15 levels are related to T‐cell immunoglobulin and immunoreceptor tyrosine‐based inhibitory motif domain expression on CD8^+^ tumour‐infiltrating lymphocytes in lung adenocarcinoma

**FIGURE 6:**
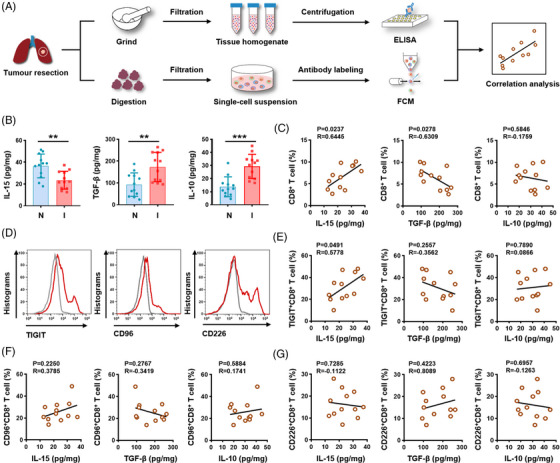
Local IL‐15 levels correlate with increased TIGIT expression on CD8^+^ TILs. (A) Flow chart of the correlation analysis among TIGIT, CD96 or CD226 expression on CD8^+^ TILs and cytokines in tissue homogenate samples. (B) Concentrations of IL‐15, TGF‐β and IL‐10 detected in tissue homogenates of normal lung and intratumoural lung (*N* = 12). (C) Correlation analysis between the concentration of IL‐15, TGF‐β, or IL‐10 and the percentage of CD8^+^ TILs. (D) Representative flow cytometry histogram showing TIGIT, CD96 or CD226 expression on CD8^+^ TILs. (E–G) Correlation analysis between the concentrations of IL‐15, TGF‐β and IL‐10 and the percentages of TIGIT^+^CD8^+^ TILs (E), CD96^+^CD8^+^ TILs (F), or CD226^+^CD8^+^ TILs (G). Results are presented as the means ± SD of four independent individuals. **P < .01, ***P < .001; two‐tailed Student's *t*‐test (B) and simple linear regression (C, E, F, G) were used. ELISA, enzyme‐linked immunosorbent assay; FCM, flow cytometry; I, intratumoural tissue; IL, interleukin; N, normal lung tissue; SD, standard deviation; TGF, transforming growth factor; TIGIT, T‐cell immunoglobulin and immunoreceptor tyrosine‐based inhibitory motif domain; TIL, tumour‐infiltrating lymphocyte.

Cytokines are essential components of the tumour microenvironment by modulating T cells’ differentiation and function, thereby manipulating tumour‐specific immunity.^17^ We conducted a comprehensive analysis using fresh LUAD tissues to investigate the relationship between local cytokine alterations and CD8^+^ TILs (Figure 6A). When comparing the tumour tissues to the healthy lung tissues, there was a notable increase in TGF‐β and IL‐10 concentrations as well as a decrease in IL‐15 levels (Figures 6B and S8), implicating an immunosuppressive microenvironment favouring tumourigenesis of LUAD. Additionally, a negative association was discovered between the concentration of TGF‐β and the percentage of CD8^+^ TILs (*R* = −.6309, *P* = .0278), but a positive correlation was detected between IL‐15 levels and the percentage of CD8^+^ TILs (*R* = .6445, *P* = .0237). No association was detected between IL‐10 levels and the percentage of CD8^+^ TILs (*R* = −.1759, *P* = .5846; Figure 6C). Subsequently, CD8^+^ TILs were categorised based on their expression of TIGIT, CD96 and CD226 (Figure 6D). The IL‐15 concentrations exhibited a significant positive correlation with the percentage of TIGIT^+^CD8^+^ TILs (*R* = .5778, *P* = .0491; Figure 6E). In addition, no significant association was found between the concentrations of the three cytokines and the percentages of CD96^+^CD8^+^ or CD226^+^CD8^+^ TIL subsets (all *P* >.05; Figure 6E–G). Altogether, these findings indicated a potential positive correlation between intratumoural IL‐15 levels and TIGIT expression on CD8^+^ TILs.

### 2.7 Interleukin‐15 in combination with T‐cell immunoglobulin and immunoreceptor tyrosine‐based inhibitory motif domain blockade enhances the activation and cytotoxicity of CD8^+^ tumour‐infiltrating lymphocytes in lung adenocarcinoma

**FIGURE 7:**
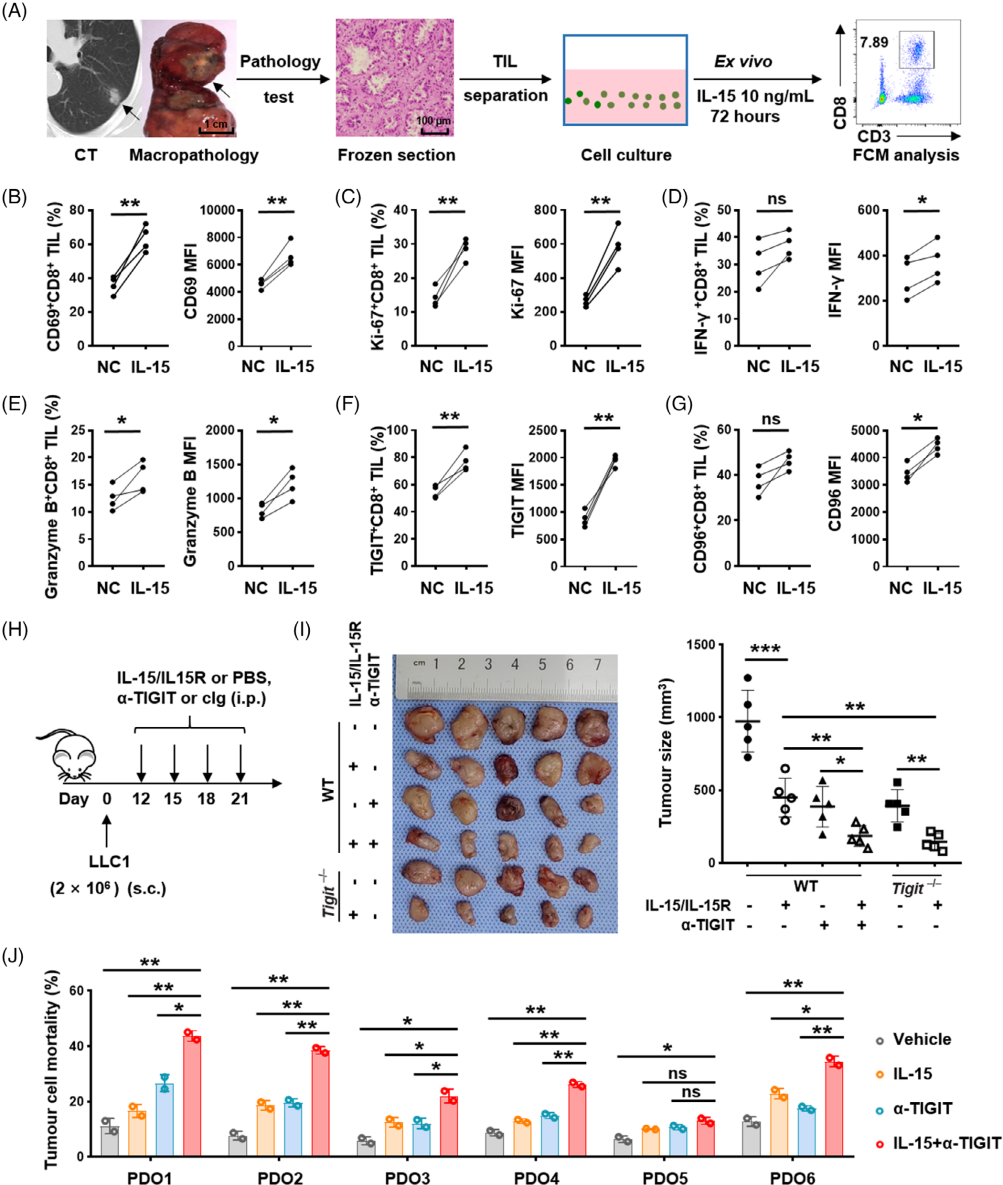
Combining IL‐15 with TIGIT blockade enhances the cytotoxicity of CD8^+^ TILs in LUAD. (A) Experimental scheme used in (B–G): TILs were separated from LUAD specimens confirmed by frozen section examination, followed by ex vitro culture with IL‐15. Representative preoperative CT, resected surgical specimen, and flow cytometry gating are shown. (B–G) Comparison of the proportions and MFI of CD69 (B), Ki‐67 (C), IFN‐γ (D), granzyme B (E), TIGIT (F) and CD96 (G) expression on CD8^+^ TILs following IL‐15 stimulation. (H) Experiment scheme: *Tigit* ^−/−^ and WT mice were inoculated s.c. with 2 × 10^6^ LLC1 cells on Day 0 and intraperitoneally (i.p.) injected with IL15/IL‐15R, anti‐TIGIT, or their combination on Days 12, 15, 18 and 21. (I) Tumour size as in (H) at Day 28 after challenge. Data are presented as the means ± SD and representative graph of two independent experiments is shown. (J) Tumour cell mortality in PDOs treated with control, IL‐15, anti‐TIGIT, or their combination following coculture with autologous CD8^+^ TILs. Data are presented as the means ± SD of two biologically parallel experiments. *P < .05, **P < .01, ns, no significant difference; two‐tailed Student's *t*‐test (B–G) and one‐way ANOVA with Tukey's post hoc test (I, J) were used. CT, computerised tomography; FCM, flow cytometry; cIg, control immunoglobulin; NC, negative control; PDO, patient‐derived organoid; TIGIT, T‐cell immunoglobulin and immunoreceptor tyrosine‐based inhibitory motif domain; TIL, tumour‐infiltrating lymphocyte; IL, interleukin; IFN, interferon; LUAD, lung adenocarcinoma; MFI, mean fluorescence intensity; WT, wild‐type; SD, standard deviation.

Considering that our findings support a positive correlation between IL‐15 and immune checkpoint expression on CD8^+^ TILs, we next set out to explore the effect of IL‐15 on CD8^+^ TILs in LUAD. CD8^+^ TILs isolated from freshly resected LUAD were cultured with 10 ng/mL IL‐15 for a duration of 72 h (Figure 7A). Through flow cytometry analysis, a notable rise was found in the levels of both CD69, an activation marker, and Ki‐67, a proliferation marker, within CD8^+^ TILs following exposure to IL‐15 (Figure 7B,C). Additionally, IL‐15 stimulation resulted in a moderate augmentation of IFN‐γ and granzyme B secretion (Figure 7D,E). These data suggest that IL‐15 exhibits a modest stimulatory effect on CD8^+^ TILs in LUAD. Interestingly, IL‐15 stimulation resulted in a significantly increased expression of TIGIT and a modestly elevated expression of CD96 on CD8^+^ TILs (Figure 7F,G), indicating that the activation induced by IL‐15 is accompanied by simultaneous upregulation of inhibitory receptors.

The overexpression of TIGIT impairs the functionality of CD8^+^ T cells, thereby partially counteracting the activation effects induced by an IL‐15 agonist. To enhance the cytotoxicity of CD8^+^ T cells, we propose a therapeutic strategy that combines TIGIT blockade with IL‐15 stimulation. In an in vitro cytotoxicity assay, the combination of α‐TIGIT and IL‐15 resulted in significantly enhanced lysis of H2228 cells compared to treatment with α‐TIGIT or IL‐15 alone (Figure S9). Furthermore, we constructed a transplanted tumour murine model to investigate the in vivo efficacy of this combination therapy (Figure 7H). Treatment with IL‐15 or anti‐TIGIT resulted in significant suppression of tumour progression; notably, treatment with IL‐15 exhibited the most significant inhibition of tumour growth in *Tigit* ^−/−^ mice (Figure 7I). To validate the efficacy of this combination therapy in tumour eradication, a patient‐derived organoid (PDO) model was established using freshly resected surgical specimens. Autologous CD8^+^ TILs, isolated from patient tumour tissues, were cocultured with the PDOs and subsequently treated with IL‐15, anti‐TIGIT, or their combination (Figure S10A,B). The combination of anti‐TIGIT and IL‐15 led to a significantly higher proportion of LUAD organoid death than inhibition of TIGIT alone or treatment with IL‐15 alone (Figures 7J and S10C,D). Altogether, these data support the utility of TIGIT blockade combined with IL‐15 stimulation in promoting autologous CD8^+^ T‐cell‐mediated immune responses against LUAD.

## 3 DISCUSSION

In recent years, the development of immunotherapy represented by PD‐1 and CTLA‐4 blockade has fundamentally changed the tumour treatment paradigm. However, as clinical data accumulate, low response rates and serious adverse effects have begun to be revealed, prompting researchers to explore novel targets within the tumour immune microenvironment.^18^ TIGIT has emerged as an attractive immune checkpoint for the cancer therapy. The upregulation of TIGIT has been reported in several types of malignancies including intestinal stomach cancer, melanoma, acute myeloid leukaemia and multiple myeloma.^11^, ^12^, ^19^, ^20^, ^21^, ^22^ However, most of those studies have ignored the dynamics of the TIGIT/CD96/CD226 cosignalling network, which is essential for regulating the immune response.^7^ In our study, an increase was observed in the TIGIT expression on peripheral T cells; importantly, TIGIT expression was significantly greater on CD8^+^ TILs in patients with LUAD, thereby tipping the balance between the three receptors. Mechanistically, IL‐15 enhanced the effector function of CD8^+^ TILs but stimulated the TIGIT expression on CD8^+^ TILs concomitantly. This suggests a potential combined immunotherapy of IL‐15 stimulation and TIGIT blockade in the treatment of LUAD.

The coinhibitory receptors TIGIT and CD96, as well as the costimulatory receptor CD226, compete for binding to the same ligand CD155, thereby fine‐tuning immune responses.^23^ Among the three receptors, TIGIT exhibits the highest affinity for CD155 followed by CD96 and CD226.^6^ Consequently, TIGIT can outcompete CD226 in binding to CD155, thus disrupting the costimulation mediated by CD226. Additionally, TIGIT may directly interact with CD226, interfering with its cis‐homodimerization and binding capacity to CD155.^11^ We observed a transition from CD8^+^ T cells expressing CD226 and CD96 to those expressing TIGIT once these cells had infiltrated into the cancer nest, consequently disrupting the immune equilibrium between the three receptors observed in healthy lungs. The immune imbalance is bound to the dysfunction of CD8^+^ T cells, which might facilitate tumour evasion of immune surveillance and promote tumour growth. The positive correlation between the dysregulated interplay among TIGIT, CD96 and CD226 expression and poor patient survival further supported this perspective. Therefore, an immune imbalance between the three receptors not only facilitates a more comprehensive delineation of prognostic features but also opens up novel avenues for the treatment of LUAD.

The tumourigenic role of TIGIT has not been extensively investigated. Given the rarity of tumour‐infiltrating Tregs and NK cells in LUAD, the assessment of TIGIT's effect on these cell populations in clinical samples was not taken into consideration. However, the inhibitory effect of TIGIT was observed specifically on the CD8^+^ T‐cell‐mediated antitumour responses in tumour‐bearing mice, rather than NK cells, CD4^+^ T cells, or Tregs. In our model, the predominant immunosuppressive effect of TIGIT was confined to CD8^+^ T cells, despite previous reports emphasizing its role in suppressing antitumour immunity through Tregs or NK cells.^24^, ^25^ Here, the immunosuppressive effects of TIGIT on CD8^+^ T cells were clarified by several lines of evidence. First, there was a significant reduction in the secretion of effector cytokines by TIGIT^+^CD8^+^ TILs upon stimulation with phorbol 12‐myristate 13‐acetate (PMA) and ionomycin. Second, transcriptomic analysis comparing TIGIT^+^CD8^+^ TIL and TIGIT^–^CD8^+^ TIL subsets revealed that genes associated with immune inhibition and exhaustion were more abundant in TIGIT^+^CD8^+^ TILs. Moreover, the treatment with anti‐TIGIT antibody significantly enhanced the cytotoxicity of CD8^+^ T cells. In line with this, blockade of TIGIT might boost CD8^+^ T‐cell‐mediated cytotoxic activity against LUAD and has the potential to be a promising target for CD8^+^ T‐cell‐mediated immunotherapy.

Although the inhibition of TIGIT alone or combined with PD‐1 blockade can effectively restore the T‐cell activity in some cases,^21^, ^26^ immune checkpoint inhibitors for the anticancer therapy always fail to fulfill expectations. Recently, combination therapies involving immune checkpoint blockade and immunoregulatory cytokines have been tested in murine models and clinical trials.^27^, ^28^ Immunoregulatory cytokines represent an essential element of signalling pathways during tumour‐related immune responses. Associations between the changes in certain circulating or intratumoural cytokines and prognosis have been identified in NSCLC patients.^29^, ^30^, ^31^, ^32^ The levels of TGF‐β and IL‐10 in the local LUAD tissue exhibited a significant increase, while there was a concurrent decrease in the intratumoural levels of IL‐15 in local LUAD tissue compared to that in adjacent normal lung tissue. Moreover, it was surprising to discover a positive correlation between IL‐15 levels and TIGIT^+^CD8^+^ TILs, suggesting that IL‐15 may regulate the function and behaviour of CD8^+^ TILs mediated by TIGIT. IL‐15, a member of IL‐2 family of cytokines, enhances T‐ and NK‐cell immune responses.^33^ IL‐15 has been tested in the clinic and showed profound effects on the expansion of T cells and NK cells. However, similar to IL‐2, systemic toxicities remained prevalent and limited clinical benefit persisted using IL‐15 as monotherapy.^34^, ^35^ Our data demonstrated that exposure to IL‐15 promoted the activation, proliferation, and slightly elevated cytotoxicity of CD8^+^ TILs in patients with LUAD, which was consistent with clinical findings regarding the capacity of IL‐15 as an agonist for CD8^+^ T cells. Interestingly, a concomitant increase in the inhibitory receptors TIGIT and CD96 was observed, which may provide insights into the limited clinical efficacy of IL‐15 monotherapy, although not exclusively attributed to it. In a transplanted tumour murine model, treatment with IL‐15 significantly suppressed tumour growth in *Tigit* ^−/−^ mice compared to WT littermates. These findings provide additional evidence supporting our speculation concerning the inhibitory role of TIGIT in limiting the therapeutic potential of IL‐15 monotherapy.

The activation and dysfunction of CD8^+^ T cells is a tightly regulated process. When exposed to immune stimulatory signals and tumour cell targets, CD8^+^ T cells undergo a spectrum of T‐cell phases ranging from functional effector to hyporesponsive.^1^ As the upregulation of TIGIT following IL‐15 stimulation may lead to the exhaustion of CD8^+^ TILs and limit the activation efficacy of IL‐15, IL‐15 therapy combined with TIGIT blockade seems to be a rational and biomarker‐driven strategy to optimise the antitumour effects of CD8^+^ T cells. Indeed, the administration of IL‐15 and anti‐TIGIT significantly increased the lysis of H2228 cells mediated by CD8^+^ TILs isolated from LUAD in comparison to IL‐15 or anti‐TIGIT alone. This combination therapy strategy has also been reported to increase NK cell‐ or CD8^+^ T‐cell‐mediated effector function in melanoma and soft tissue sarcomas.^36^, ^37^ However, these studies have been limited to cell lines or murine models and cannot accurately replicate the immune environment of tumours. Our study demonstrated that this combination therapy strategy could promote the cytotoxicity of autologous CD8^+^ TILs in a LUAD organoids model. This suggests that IL‐15 stimulation in combination with TIGIT blockade can greatly augment the activity of preexisting tumour antigen‐specific CD8^+^ T cells and promote tumour‐specific immune responses. Given the rarity of tumour‐infiltrating cells and the scarcity of clinical samples, we could not reach any conclusions concerning the effects of combined treatment on NK cells or other lymphocytes. The administration of IL‐15 and anti‐TIGIT was tested using a transplanted tumour murine model and PDO model, which provided strong support for this conclusion. The mechanisms by which IL‐15 and TIGIT blockade reinvigorate CD8^+^ T‐cell‐mediated killing of LUAD cells remain to be thoroughly investigated.

**FIGURE 8:**
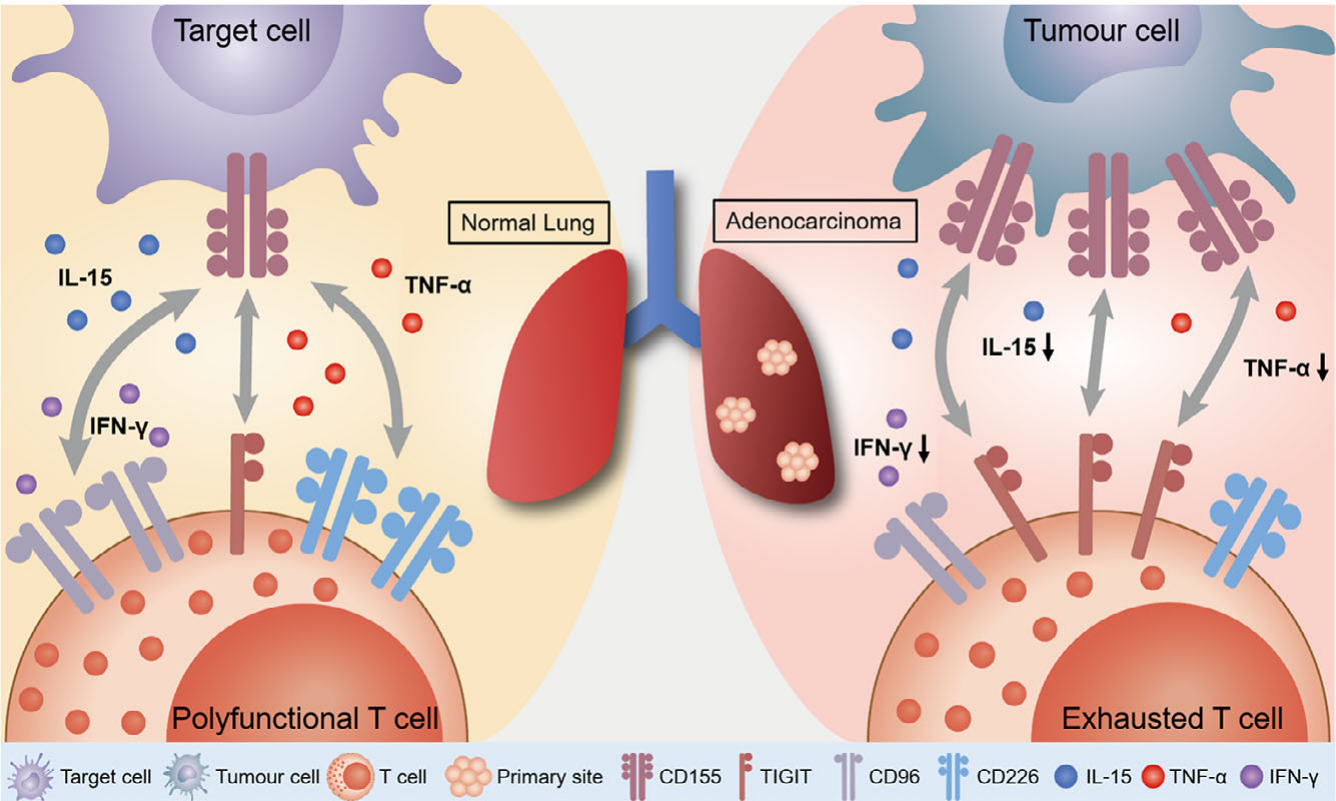
The expression of coinhibitory receptors TIGIT and CD96, as well as costimulatory receptor CD226 on CD8^+^ T cells is dysregulated in LUAD, contributing to immune evasion by tumour cells. The proposed mechanism suggests a transition from CD226 and CD96 to TIGIT within the tumour microenvironment. Elevated levels of TIGIT inhibit cytokine production and the cytotoxic activities of CD8^+^ T cells, thereby promoting tumour progression. Moreover, the expression of TIGIT on CD8^+^ T cells in LUAD was regulated by IL‐15. It is anticipated that combining TIGIT blockade with IL‐15 stimulation could enhance the antitumour immunity of CD8^+^ T cells. TIGIT, T‐cell immunoglobulin and immunoreceptor tyrosine‐based motif inhibitory; IL, interleukin; IFN, interferon; TNF, tumour necrosis factor; LUAD, lung adenocarcinoma.

In summary, the present study reveals a dynamic immune imbalance between the expression of TIGIT, CD96 and CD226 on CD8^+^ T cells (Figure 8). Dysregulation of TIGIT induced the dysfunction of CD8^+^ TILs and suppressed antitumour immunity. Furthermore, there is a correlation between TIGIT expression on CD8^+^ TILs and IL‐15 levels, with IL‐15 stimulation leading to the activation of CD8^+^ TILs and concurrent upregulation of TIGIT. Notably, IL‐15 stimulation combined with TIGIT blockade reinvigorates CD8^+^ TIL‐mediated antitumour immunity in tumour‐bearing mice and LUAD organoids. Altogether, our data identify TIGIT as a promising therapeutic target and pave the way for combination immunotherapy using IL‐15 with TIGIT blockade for the treatment of LUAD.

## 4 METHODS

### 4.1 Lung adenocarcinoma tissue and blood samples

All LUAD paraffin tissue samples were obtained from the Department of Pathology at the First Affiliated Hospital of Sun Yat‐sen University. Fresh surgical specimens were collected within 30 min after surgical resection. Single‐cell suspensions for flow cytometry analysis were prepared after digestion and filtration. Blood specimens were obtained intravenously and separated using Percoll solution, with PBMCs collected for further analysis. The comprehensive clinical characteristics are provided in Table S3. All patients underwent standard histological assessment for the diagnosis of LUAD. Prior to the surgical procedures, no additional antitumour therapies were administered. Clinical staging was determined in accordance with the guidelines established by the International Union Against Cancer.

### 4.2 Cell lines

The H2228 and LLC1 cell lines were obtained from ATCC and confirmed by STR profiling. The H2228 cells overexpression CD155 were generated by infecting H2228 cells with recombinant lentiviruses carrying the CD155‐IRES‐RFP moiety and subsequently sorting RFP^high^ cells. The infection efficiency was further confirmed through quantitative PCR and western blot analysis. Routine testing for mycoplasma contamination was regularly conducted on the cell lines.

### 4.3 Immunohistochemistry

The paraffin sections were deparaffinised and subsequently underwent antigen retrieval, peroxidase blocking, protein blocking, antibody incubation and staining according to standardised procedures. The following antibodies for immunohistochemical staining were used: anti‐CD155 (D8A5G, Cell Signaling Technology), anti‐CD96 (BLR065G, Abcam), anti‐CD226 (E8L9G, Cell Signaling Technology), anti‐TIGIT (BLR047F, Abcam), anti‐CD3 (LN10, Zsbio), anti‐CD4 (UMAB64, Zsbio), anti‐CD8 (SP16, Zsbio), anti‐CD56 (UMAB83, Zsbio), anti‐CD25 (EPR6452, Abcam) and anti‐Foxp3 (236A/E7, Abcam). Images of immunohistochemical staining were captured using a microscope (Leica). The immunoscores were evaluated in a double‐blind manner by two pathologists. The staining proportion was assessed on a scale of five gradations: 0 (0%–9%), 1 (10%–25%), 2 (26%–50%), 3 (51%–75%) or 4 (76%–100%). The staining intensity was classified into four levels as follows: 0 (absent staining), 1 (pale brown), 2 (moderate brown) or 3 (dark brown). The cumulative immunoscore was calculated by multiplying the staining proportion and intensity, ranging from 0 to 12.

### 4.4 Separation of tumour‐infiltrating lymphocytes

Fresh LUAD tissue specimens were digested and homogenised with PBS in C tubes (Miltenyi) using a gentleMACS Dissociator (Miltenyi). The solutions underwent filtration through a 100 μm mesh filter and centrifugation using the Percoll separation solution (TBD Science). The interface layer cells were collected for the separation of CD8^+^ T cells using IMag anti‐human CD8 Particles (BD).

### 4.5 Flow cytometry

For analysis of surface markers, the single‐cell suspensions of PBMCs or TILs were labelled with fluorochrome‐conjugated monoclonal antibodies specific to the relevant targets for 30 min. The primary antibodies include: anti‐human PE/cy7‐CD3 (OKT3, eBioscience), FITC‐CD4 (OKT4, eBioscience), Alexa Fluor 700‐CD8 (HIT8a, BD), Brilliant Violet 421‐TIGIT (MBSA43, eBioscience), APC‐CD96 (NK92.39, eBioscience), PE‐CD226 (c11A8, BioLegend) and FITC‐CD69 (FN50, BioLegend). For the detection of intracellular markers, cells were cultured with PMA/ionomycin/brefeldin A (eBioscience) for 4 h and then were permeabilised using a fixation/permeabilization kit (eBioscience). The primary antibodies include: anti‐human APC/CY7‐IFN‐γ (4S.B3, eBioscience), Alexa Fluor 647‐TNF‐α (MAB11, eBioscience), Pacific Blue‐granzyme B (QA16A02, BioLegend), and PE‐Ki‐67 (Ki‐67, BioLegend). For mouse cell staining, the primary antibodies include: anti‐mouse APC/cy7‐CD45 (30‐F11, BioLegend), FITC‐CD3 (17A2, BioLegend), PE‐CD4 (GK1.5, BioLegend), Alexa Fluor 700‐CD8 (Mp6‐XT22, BioLegend), PE/cy7‐CD69 (H1.2F3, eBioscience), PE‐IFN‐γ (XMG1.2, eBioscience), Brilliant Violet 421‐TNF‐α (Mp6‐XT22, BioLegend), APC‐IL‐2(JES6‐5H4, BioLegend), APC‐NK1.1 (S17016D, BioLegend) and BV421‐Foxp3 (MF‐14, eBioscience).

### 4.6 Cytotoxicity assay

A standard 4‐h carboxyfluorescein diacetate succinimidyl ester (CFSE)‐based assay was used to assess the cytotoxicity of CD8^+^ T cells against H2228 cells. Briefly, H2228 cells were labelled with 10 μM CFSE (eBioscience) and then seeded onto plates as target cells. Subsequently, CD8^+^ T cells were isolated and exposed to H2228 cells at various E:T ratios. After incubation in the presence of blocking antibodies for 4 h, the cells were stained with 7‐AAD (eBioscience) followed by flow cytometry analysis. The blocking antibodies used were as follows: anti‐CD155 antibody (D171, GeneTex), anti‐TIGIT antibody (MBSA43, eBioscience), anti‐CD96 antibody (6A6, eBioscience) and control IgG (BD Pharmingen).

### 4.7 Genome‐wide transcriptional profiling

TILs were isolated from LUAD tissues through human CD8 MicroBeads (Miltenyi) separation and flow cytometry sorting. Using MiniBEST Universal RNA Extraction Kit (TaKaRa), RNA was extracted. The quality of the extracted RNA was assessed by Qubit 3.0 (Invitrogen). The TruSeq mRNA Library Preparation Kit (Illumina) was used to construct an RNA‐Seq library. Sequencing services were provided by Lc‐Bio Technologies (China). The cutoff criteria for the differential gene expression analysis was FDR values < .05 and log2‐fold‐change (FC) ≥ 1. After *z*‐score normalization, the gene expression profile was visualised in a heatmap format by OmicStudio tools.

### 4.8 RT‐qPCR

The mRNA was separately extracted from the collected TIGIT^+^CD8^+^ TILs and TIGIT^–^CD8^+^ TILs using RNAex Pro Reagent (AG, USA). Relative quantification of mRNA expression was performed using the SYBR Green Premix qPCR Kit (AG, USA). The following primer pairs were used: KLRG1, forward 5′‐TGA CAG TGT TAT TTA TTC CAT GTT AGA G‐3′ and reverse 5′‐CCT CCT TTT AGG GAT ACA TG‐3′; GZMB, forward 5′—GGG CAG ATG CAG ACT TTT CC‐3′ and reverse 5′‐ GGC CCC CAA GGT GAC ATT TA‐3′; PRF1, forward 5′‐ TGG GTG GTC TAA GAG CCC TG‐3′ and reverse 5′‐ CAC TGG GCA CTC CCA GAT TT‐3′; and PDCD1, forward 5′‐TTT CAG GAA TGG GTT CCA AG‐3′ and reverse 5′‐ACA TCC TAC GGT CCC AAG GT‐3′.

### 4.9 Transplanted tumour murine model

*Tigit* ^−/−^ mice were gifts from Professor Xingxu Huang of Shanghai Tech University and the knockout of TIGIT did not alter the immune homeostasis of the mice.^38^ *Tigit* ^−/−^ and litter WT mice (8–10 weeks, females) were allocated randomly into different groups, each consisting of five *Tigit* ^−/−^ and five C57BL/6 WT mice. Mice in these groups were inoculated subcutaneously (s.c.) with LLC1 cells and intraperitoneally (i.p.) injected with antibodies or cytokines. The depletion antibodies used were as follows: anti‐asialoGM1 (Poly21460, BioLegend), anti‐CD8β (35‐17.2, BioLegend), anti‐CD4 (GK1.5, BioLegend), anti‐CD25 (7D4/CD25, BioLegend) or control immunoglobulin (cIg, BioLegend). The blockade antibodies were as follows: anti‐TIGIT (Bioxcell, BE0274) or control immunoglobulin G (IgG, BioLegend). Cytokines were as follows: murine IL‐15 (Biolegend), recombinant IL‐15Rα‐Fc (Novoprotein). All mice were observed daily for any abnormalities after inoculation. Tumour size was measured and recorded daily after subcutaneous tumour formation. Mice were euthanised when one of the following conditions occurred: severe systemic infection, significant weight loss or obvious endangerment manifestations.

### 4.10 Multicolour immunofluorescence staining

Multicolour immunofluorescence staining was performed on FFPE tissues using Opal 7‐Colour Manual IHC Kit (NEL811001KT). In brief, the sections underwent xylene deparaffinisation and rehydration followed by epitope retrieval. At each staining cycle, following a 10‐min incubation with blocking solution, the sections were incubated for 1 h with primary antibodies including: anti‐CD4 (Abcam, EPR6855, 1:400), anti‐CD25 (Abcam, EPR6452, 1:100), anti‐FoxP3 (R&D, 1054C, 1:500), PanCK (Biolynx, BP6051/BP6058, 1:1000), TTF‐1 (Invitrogen, 2F4D8, 1:200) or CK7 (Proteintech, Polyclonal antibody, 1:200). Then, the sections were incubated with horse radish peroxidase (HRP)‐conjugated secondary antibody (GeneTech) for 15 min. Tyramide signal amplification (TSA) fluorophores were further incubated for 10 min to amplify the signal. Lastly, DAPI counterstaining for 5 min was performed. PerkinElmer Vectra (PerkinElmer, CLS140089) was used to scan the slides and InForm Advanced Image Analysis Software (PerkinElmer) was used to analyse the multispectral images.

### 4.11 Organoid culture

Single cells were isolated from fresh LUAD tissues and suspended in DMEM/F12 medium (Gibco) containing with 10% Matrigel (Corning). After Matrigel was solidified, the human organoid medium (bioGenous) was added, and the microplate was cultured at 37°C in a humidified atmosphere with 5% CO_2_. The growth status and morphology of tumour spheroids were observed using an inverted microscope every day. Approximately once a week, organoids were digested in Tryple Express (Gibco) and passaged in fresh Matrigel.

### 4.12 Organoid killing assays

Autologous CD8^+^ TILs were isolated from LUAD tissues and cultured for 72 h on plates coated with anti‐CD28 (37.51, eBioscience). Tumour organoids were harvested when their average diameters exceeded 50 μm. A portion of the organoid spheroids was digested into single cells for cell counting. The remaining organoid spheroids were cocultured with autologous CD8^+^ TILs at an E:T ratio of 20:1 in the presence of IL‐15 (R&D Systems), anti‐TIGIT antibody (MBSA43, eBioscience), their combination, or control IgG (BD Pharmingen). After 72 h of coculture, the organoids were dissociated into single cells using TrypLE Express and stained with 7‐AAD (eBioscience) for further flow cytometry analysis. To assess the time‐dependent killing of organoids, a green‐fluorescent probe specific for caspase 3/7 (Invitrogen), diluted at a ratio of 1:2000, was introduced into the coculture system to monitor apoptotic processes. The efficacy of T cell‐mediated cytotoxicity was evaluated over a 24‐h period using the LionheartFX, a live‐cell real‐time fluorescence imaging system developed by Biotek.

### 4.13 Statistical analyses

GraphPad Prism 9.0 was used to perform statistical analyses. Kruskal‒Wallis nonparametric tests and multiple comparisons were applied to analyse TIGIT, CD96 and CD226 expression in lung tissues. The overall survival was assessed using the Kaplan‒Meier survival curve with log‐rank test, univariate analysis, and multivariate Cox proportional hazard analysis. The correlation coefficients were determined by simple linear regression with Pearson correlation analysis. Additional comparison tests include two‐tailed Student's *t*‐test, one‐way and two‐way ANOVA with Tukey's post hoc test, and Welch's *t*‐test. Data are expressed as mean ± standard deviation (SD). A *P*‐value less than.05 was used to evaluate the statistical significance.

## AUTHOR CONTRIBUTIONS

Baohong Luo, Yu Sun and Qinru Zhan designed and performed all the experiments and analysed the data. Yuting Luo, Yu Chen, Lijuan Ren, Zhongpeng Xie and Bixia Liu provided clinical samples and conducted pathological evaluation. Tongze Fu, Tiantian Yang, and Xiaohua Situ performed the bioinformatics analysis. Zunfu Ke and Kejing Tang conceptualised and initiated the project and wrote the manuscript. All authors approved the submitted version.

## CONFLICT OF INTEREST STATEMENT

The authors declare no conflicts of interest.

## ETHICS STATEMENT

The use of clinical specimens and animal experiments were ethically approved by the hospital's Clinical Research and Laboratory Animal Ethics Committee. All patients provided informed consent forms.

## Supporting information

Supporting Information

## Data Availability

The corresponding author can provide the data and materials used in the current study upon reasonable request.

## References

[1] Philip M , Schietinger A . CD8(+) T cell differentiation and dysfunction in cancer. Nat Rev Immunol. 2022;22:209‐223. doi:10.1038/s41577-021-00574-3 34253904 PMC9792152

[2] Brambilla E , Le Teuff G , Marguet S , et al. Prognostic effect of tumor lymphocytic infiltration in resectable non‐small‐cell lung cancer. J Clin Oncol. 2016;34:1223‐1230. doi:10.1200/jco.2015.63.0970 26834066 PMC4872323

[3] O'brien SM , Klampatsa A , Thompson JC , et al. Function of human tumor‐infiltrating lymphocytes in early‐stage non‐small cell lung cancer. Cancer Immunol Res. 2019;7:896‐909. doi:10.1158/2326-6066.Cir-18-0713 31053597 PMC6548666

[4] Zhou F , Qiao M , Zhou C . The cutting‐edge progress of immune‐checkpoint blockade in lung cancer. Cell Mol Immunol. 2021;18:279‐293. doi:10.1038/s41423-020-00577-5 33177696 PMC8027847

[5] Garon EB , Rizvi NA , Hui R , et al. Pembrolizumab for the treatment of non‐small‐cell lung cancer. N Engl J Med. 2015;372:2018‐2028. doi:10.1056/NEJMoa1501824 25891174

[6] Yu X , Harden K , C Gonzalez L , et al. The surface protein TIGIT suppresses T cell activation by promoting the generation of mature immunoregulatory dendritic cells. Nat Immunol. 2009;10:48‐57. doi:10.1038/ni.1674 19011627

[7] Dougall WC , Kurtulus S , Smyth MJ , Anderson AC . TIGIT and CD96: new checkpoint receptor targets for cancer immunotherapy. Immunol Rev. 2017;276:112‐120. doi:10.1111/imr.12518 28258695

[8] Wu B , Zhong C , Lang Qi , et al. Poliovirus receptor (PVR)‐like protein cosignaling network: new opportunities for cancer immunotherapy. J Exp Clin Cancer Res. 2021;40:267. doi:10.1186/s13046-021-02068-5 34433460 PMC8390200

[9] Chauvin J‐M , Zarour HM . TIGIT in cancer immunotherapy. J Immunother Cancer. 2020;8:e000957. doi:10.1136/jitc-2020-000957 32900861 PMC7477968

[10] Rodriguez‐Abreu D , Johnson ML , Hussein MA , et al. Primary analysis of a randomized, double‐blind, phase II study of the anti‐TIGIT antibody tiragolumab (tira) plus atezolizumab (atezo) versus placebo plus atezo as first‐line (1L) treatment in patients with PD‐L1‐selected NSCLC (CITYSCAPE). J Clin Oncol. 2020;38:9503‐9503. doi:10.1200/JCO.2020.38.15_suppl.9503

[11] Johnston RJ , Comps‐Agrar L , Hackney J , et al. The immunoreceptor TIGIT regulates antitumor and antiviral CD8(+) T cell effector function. Cancer Cell. 2014;26:923‐937. doi:10.1016/j.ccell.2014.10.018 25465800

[12] Sun Yu , Luo J , Chen Y , et al. Combined evaluation of the expression status of CD155 and TIGIT plays an important role in the prognosis of LUAD (lung adenocarcinoma). Int Immunopharmacol. 2020;80:106198. doi:10.1016/j.intimp.2020.106198 31954274

[13] Calì B , Molon B , Viola A . Tuning cancer fate: the unremitting role of host immunity. Open Biol. 2017;7:170006. doi:10.1098/rsob.170006 28404796 PMC5413907

[14] Blake SJ , Dougall WC , Miles JJ , Teng MWL , Smyth MJ . Molecular pathways: targeting CD96 and TIGIT for cancer immunotherapy. Clin Cancer Res. 2016;22:5183‐5188. doi:10.1158/1078-0432.Ccr-16-0933 27620276

[15] Kirilovsky A , Marliot F , El Sissy C , Haicheur N , Galon J , Pagès F . Rational bases for the use of the immunoscore in routine clinical settings as a prognostic and predictive biomarker in cancer patients. Int Immunol. 2016;28:373‐382. doi:10.1093/intimm/dxw021 27121213 PMC4986234

[16] Kučan Brlić P , Lenac Roviš T , Cinamon G , Tsukerman P , Mandelboim O , Jonjić S . Targeting PVR (CD155) and its receptors in anti‐tumor therapy. Cell Mol Immunol. 2019;16:40‐52. doi:10.1038/s41423-018-0168-y 30275538 PMC6318332

[17] Li L , Yu R , Cai T , et al. Effects of immune cells and cytokines on inflammation and immunosuppression in the tumor microenvironment. Int Immunopharmacol. 2020;88:106939. doi:10.1016/j.intimp.2020.106939 33182039

[18] Passaro A , Brahmer J , Antonia S , Mok T , Peters S . Managing resistance to immune checkpoint inhibitors in lung cancer: treatment and novel strategies. J Clin Oncol. 2022;40:598‐610. doi:10.1200/jco.21.01845 34985992

[19] Liang R , Zhu X , Lan T , et al. TIGIT promotes CD8(+)T cells exhaustion and predicts poor prognosis of colorectal cancer. Cancer Immunol Immunother. 2021;70:2781‐2793. doi:10.1007/s00262-021-02886-8 33634371 PMC10992182

[20] Lee WJ , Lee YeJ , Choi ME , et al. Expression of lymphocyte‐activating gene 3 and T‐cell immunoreceptor with immunoglobulin and ITIM domains in cutaneous melanoma and their correlation with programmed cell death 1 expression in tumor‐infiltrating lymphocytes. J Am Acad Dermatol. 2019;81:219‐227. doi:10.1016/j.jaad.2019.03.012 30880064

[21] Guillerey C , Harjunpää H , Carrié N , et al. TIGIT immune checkpoint blockade restores CD8(+) T‐cell immunity against multiple myeloma. Blood. 2018;132:1689‐1694. doi:10.1182/blood-2018-01-825265 29986909

[22] Kong Y , Zhu L , Schell TD , et al. T‐Cell immunoglobulin and ITIM domain (TIGIT) associates with CD8+ T‐Cell exhaustion and poor clinical outcome in AML patients. Clin Cancer Res. 2016;22:3057‐3066. doi:10.1158/1078-0432.Ccr-15-2626 26763253

[23] Harjunpää H , Guillerey C . TIGIT as an emerging immune checkpoint. Clin Exp Immunol. 2020;200:108‐119. doi:10.1111/cei.13407 31828774 PMC7160651

[24] Kurtulus S , Sakuishi K , Ngiow S‐F , et al. TIGIT predominantly regulates the immune response via regulatory T cells. J Clin Invest. 2015;125:4053‐4062. doi:10.1172/jci81187 26413872 PMC4639980

[25] Zhang Q , Bi J , Zheng X , et al. Blockade of the checkpoint receptor TIGIT prevents NK cell exhaustion and elicits potent anti‐tumor immunity. Nat Immunol. 2018;19:723‐732. doi:10.1038/s41590-018-0132-0 29915296

[26] Chauvin J‐M , Pagliano O , Fourcade J , et al. TIGIT and PD‐1 impair tumor antigen‐specific CD8⁺ T cells in melanoma patients. J Clin Invest. 2015;125:2046‐2058. doi:10.1172/jci80445 25866972 PMC4463210

[27] Wrangle JM , Velcheti V , Patel MR , et al. ALT‐803, an IL‐15 superagonist, in combination with nivolumab in patients with metastatic non‐small cell lung cancer: a non‐randomised, open‐label, phase 1b trial. Lancet Oncol. 2018;19:694‐704. doi:10.1016/s1470-2045(18)30148-7 29628312 PMC6089612

[28] Villena‐Vargas J , Cruz TD , Markowitz G , et al. OA09.05 neoadjuvant IL‐15‐PDL1 antibody promotes T cell memory and decreases metastatic recurrence in resectable NSCLC. J Thorac Oncol. 2022;17:S26‐S27. doi:10.1016/j.jtho.2022.07.050

[29] Barrera L , Montes‐Servín E , Barrera A , et al. Cytokine profile determined by data‐mining analysis set into clusters of non‐small‐cell lung cancer patients according to prognosis. Ann Oncol. 2015;26:428‐435. doi:10.1093/annonc/mdu549 25467015

[30] Sanmamed MF , Perez‐Gracia JL , Schalper KA , et al. Changes in serum interleukin‐8 (IL‐8) levels reflect and predict response to anti‐PD‐1 treatment in melanoma and non‐small‐cell lung cancer patients. Ann Oncol. 2017;28:1988‐1995. doi:10.1093/annonc/mdx190 28595336 PMC5834104

[31] Silva EM , Mariano VS , Pastrez PRA , et al. High systemic IL‐6 is associated with worse prognosis in patients with non‐small cell lung cancer. PLoS One. 2017;12:e0181125. doi:10.1371/journal.pone.0181125 28715437 PMC5513446

[32] Safi S , Yamauchi Y , Hoffmann H , et al. Circulating interleukin‐4 is associated with a systemic T cell response against tumor‐associated antigens in treatment‐naïve patients with resectable non‐small‐cell lung cancer. Cancers. 2020;12:3496. doi:10.3390/cancers12123496 33255425 PMC7761081

[33] Pilipow K , Roberto A , Roederer M , Waldmann TA , Mavilio D , Lugli E . IL15 and T‐cell stemness in T‐cell‐based cancer immunotherapy. Cancer Res. 2015;75:5187‐5193. doi:10.1158/0008-5472.Can-15-1498 26627006 PMC4681597

[34] Conlon KC , Lugli E , Welles HC , et al. Redistribution, hyperproliferation, activation of natural killer cells and CD8 T cells, and cytokine production during first‐in‐human clinical trial of recombinant human interleukin‐15 in patients with cancer. J Clin Oncol. 2015;33:74‐82. doi:10.1200/jco.2014.57.3329 25403209 PMC4268254

[35] Conlon KC , Potter EL , Pittaluga S , et al. IL15 by continuous intravenous infusion to adult patients with solid tumors in a phase I trial induced dramatic NK‐cell subset expansion. Clin Cancer Res. 2019;25:4945‐4954. doi:10.1158/1078-0432.Ccr-18-3468 31142503 PMC6697593

[36] Judge SJ , Darrow MA , Thorpe SW , et al. Analysis of tumor‐infiltrating NK and T cells highlights IL‐15 stimulation and TIGIT blockade as a combination immunotherapy strategy for soft tissue sarcomas. J Immunother Cancer. 2020;8:e001355. doi:10.1136/jitc-2020-001355 33158916 PMC7651745

[37] Chauvin J‐M , Ka M , Pagliano O , et al. IL15 stimulation with TIGIT blockade reverses CD155‐mediated NK‐cell dysfunction in melanoma. Clin Cancer Res. 2020;26:5520‐5533. doi:10.1158/1078-0432.Ccr-20-0575 32591463 PMC8045409

[38] Chen B , Ye B , Li M , et al. TIGIT deficiency protects mice from DSS‐induced colitis by regulating IL‐17A‐producing CD4(+) tissue‐resident memory T cells. Front Immunol. 2022;13:931761. doi:10.3389/fimmu.2022.931761 35844584 PMC9283574

