# Supplementary material for: Combining TIGIT blockade with IL‐15 stimulation is a promising immunotherapy strategy for lung adenocarcinoma
Source: Clin Transl Med. 2024 Jan 27;14(1):e1553. doi: 10.1002/ctm2.1553 (PMC10819095; doi:10.1002/ctm2.1553)
Supplement: Supplementary file 1 — Supporting Information [file CTM2-14-e1553-s001.docx]

**Supporting Information**

**Combining TIGIT blockade with IL-15 stimulation is a promising immunotherapy strategy for lung adenocarcinoma**

| **Contents** | **Page** |
| --- | --- |
| *Supplementary Figure 1* | 2 |
| *Supplementary Figure 2* | 3 |
| *Supplementary Figure 3* | 3 |
| *Supplementary Figure 4* | 5 |
| *Supplementary Figure 5* | 6 |
| *Supplementary Figure 6* | 7 |
| *Supplementary Figure 7* | 8 |
| *Supplementary Figure 8* | 8 |
| *Supplementary Figure 9* | 9 |
| *Supplementary Figure 10* | 10 |
| *Supplementary Table 1* | 11 |
| *Supplementary Table 2* | 12 |
| *Supplementary Table 3* | 13 |


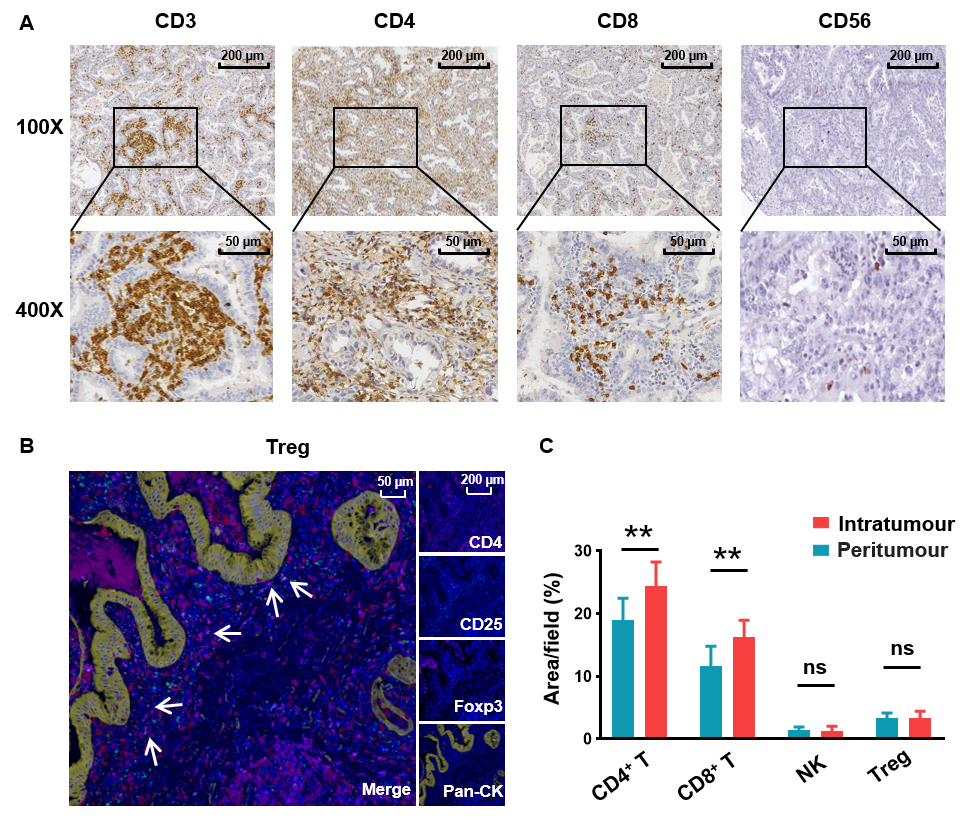


**Supplementary Figure 1** Landscape of CD4^+^ T cells, CD8^+^ T cells, NK cells, and Treg cells in patients with LUAD. **(A)** Representative IHC images of tumour-infiltrating CD4^+^ T cells, CD8^+^ T cells, and NK cells in LUAD. **(B)** Representative immunofluorescence image showing tumour-infiltrating Tregs (CD4^+^CD25^+^foxp3^+^). **(C)** Area/field of CD4^+^ T cells, CD8^+^ T cells, NK cells, and Tregs in peritumoural and intratumoural tissues of LUAD patients. Results are presented as the means ± SD and data shown are representative of 186 patients. ***P* < .01, ns, no significant difference; one-way ANOVA with Tukey’s post hoc test was used. NK, natural killer cell; Treg, regulatory T cell; LUAD, lung adenocarcinoma; IHC, immunohistochemistry; SD, standard deviation.


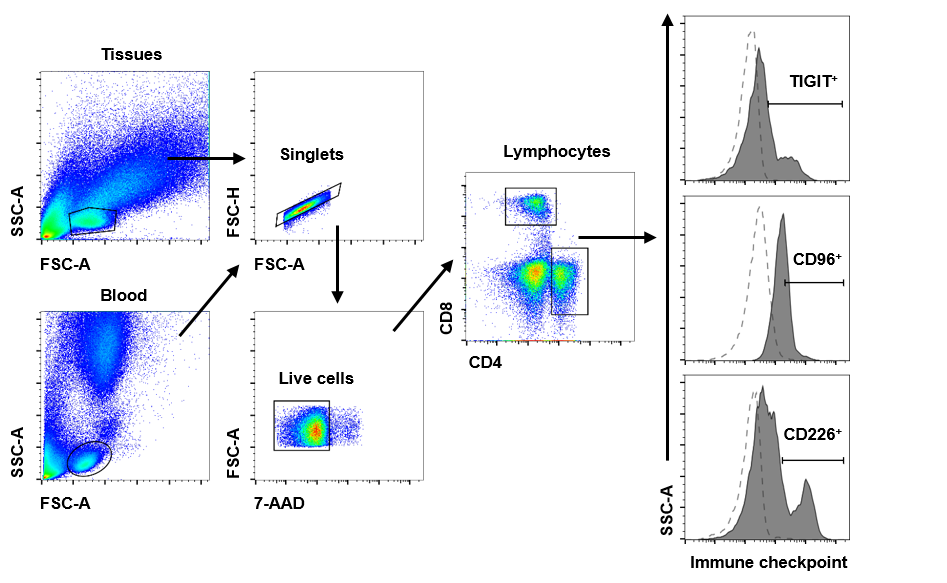


**Supplementary Figure 2** Representative flow plots of the expression of TIGIT, CD96, and CD226 (gated on CD4^+^ or CD8^+^ T cells).


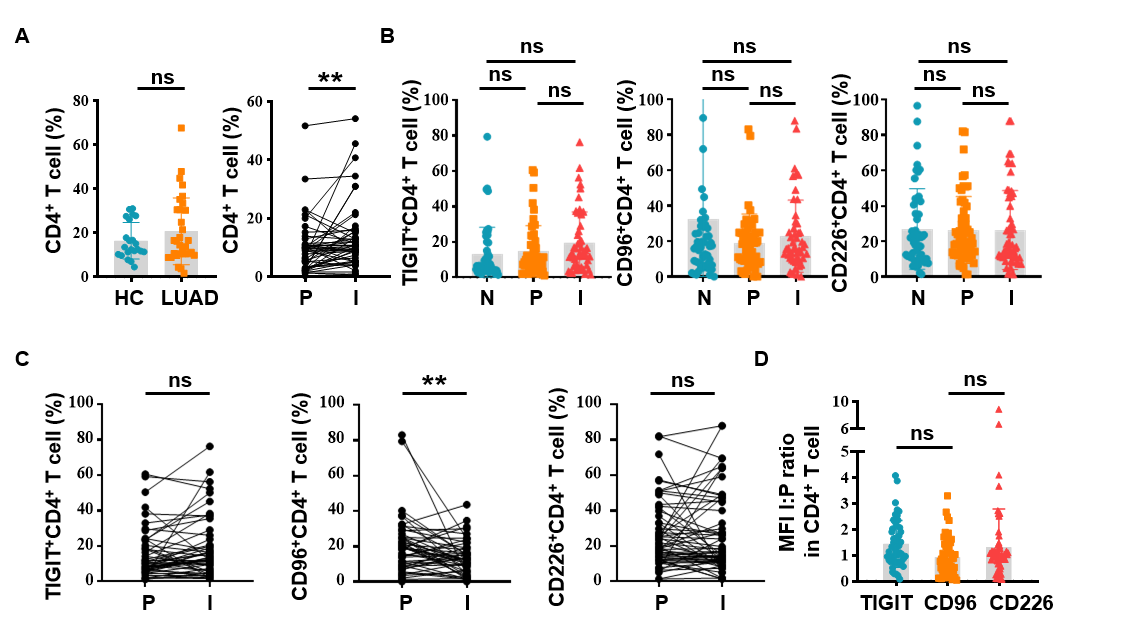


**Supplementary Figure 3** The expression of TIGIT/CD96/CD226 on CD4^+^ T cells in LUAD patients was not significantly different. **(A)** Cumulative percentage of peripheral CD4^+^ T cells in HCs and LUAD patients **(left)** and in paired peritumoural and intratumoural tissue of each LUAD patient **(right)**. **(B)** Cumulative percentage of TIGIT^+^CD4^+^ T cells, CD96^+^CD4^+^ T cells, or CD226^+^CD4^+^ T cells in normal lung, peritumoural and intratumoural lung tissues. **(C)** Cumulative percentage of TIGIT^+^CD4^+^ T cells, CD96^+^CD4^+^ T cells, or CD226^+^CD4^+^ T cells in paired peritumoural and intratumoural tissue of each LUAD patient. **(D)** The relative MFI fold-change of TIGIT, CD96, or CD226 in intratumoural CD4^+^ T cells compared to that in paired peritumoural CD4^+^ T cells. Results are presented as the means ± SD of 22-50 independent individuals. **P* < .05, ***P* < .01, ns, no significant difference; Welch's *t*-test (A left), Wilcoxon matched-pairs signed-rank test (A right), Kruskal‒Wallis ANOVA followed by Dunn’s multiple comparisons test (B, D) and Wilcoxon matched-pairs signed-rank test (C) were used. HC, healthy control; LUAD, lung adenocarcinoma; P, peritumoural tissue; I, intratumoural tissue; N, normal lung tissue; MFI, mean fluorescence intensity; SD, standard deviation..


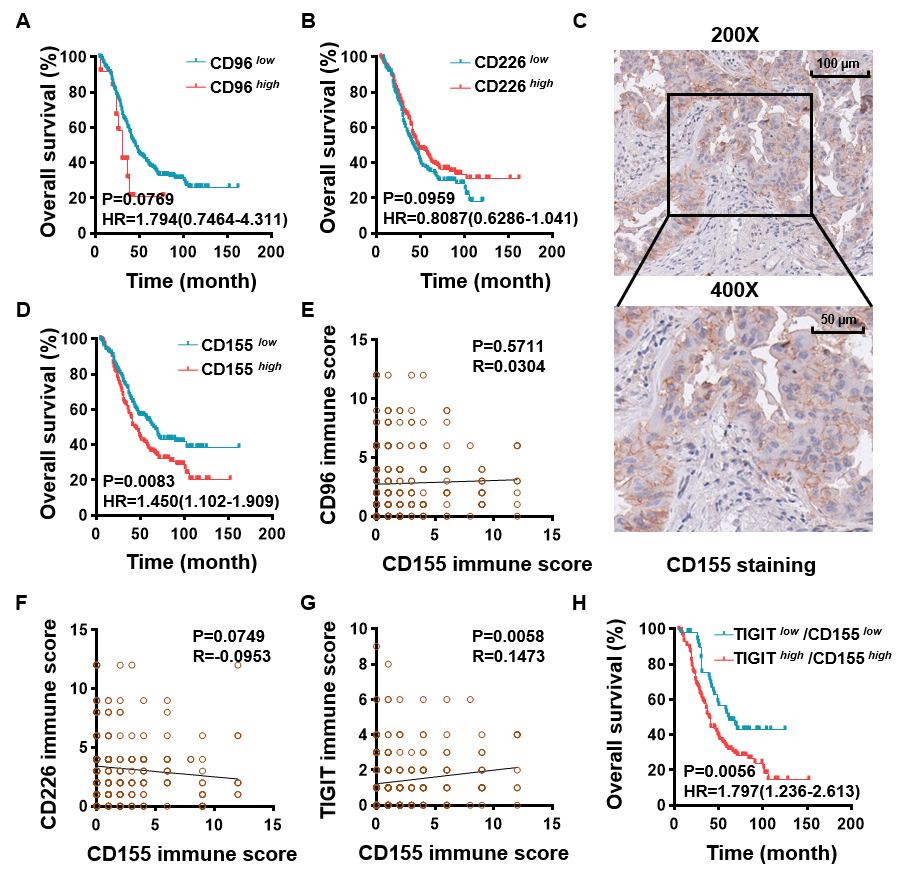


**Supplementary Figure 4** Higher CD155 score or increased density of TIGIT^+^CD155^+^ is associated with unfavorable clinical outcomes. **(A, B)** Kaplan‒Meier survival curves for overall survival according to the level of intratumoural CD96 **(A)** or CD226 **(B)** expression. **(C)** Representative microscopic images showing CD155 immunostaining in tumour cells from LUAD. Original magnifications: ×200 (top) and ×400 (bottom). **(D)** Kaplan‒Meier survival curves for overall survival according to the level of CD155 expression in tumour cells. **(E-G)** Correlation analysis between CD155 immune scores and CD96 immune scores **(E)**, or CD226 immune scores **(F)**, or TIGIT immune scores **(G)**. **H** Kaplan‒Meier survival curves for overall survival according to the density of intratumoural TIGIT^+^CD155^+^. Log-rank test (A, B, D, H) and simple linear regression (E, F, G) were used.


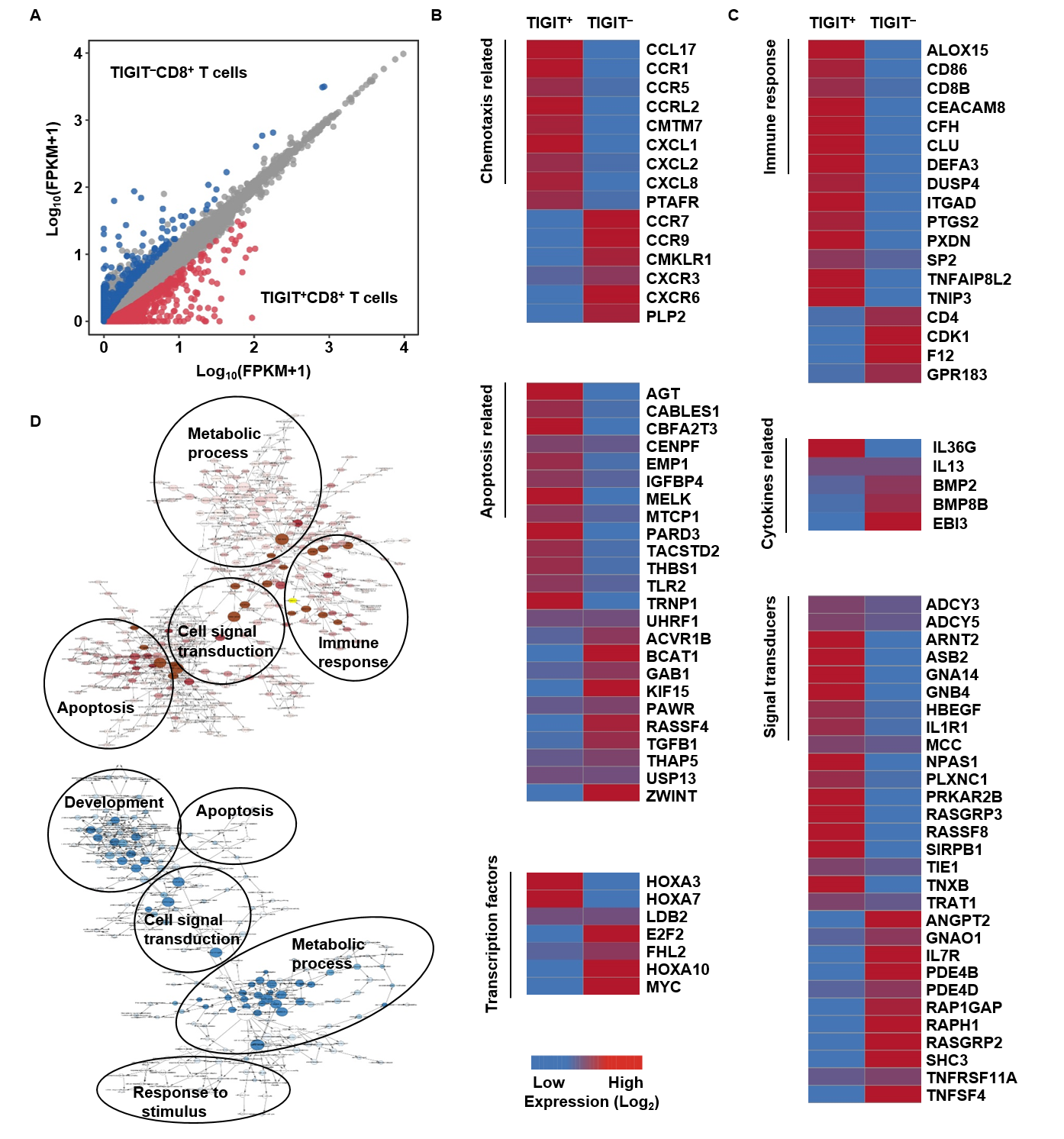


**Supplementary Figure 5** Comparison between TIGIT^+^CD8^+^ TIL and TIGIT^–^CD8^+^ TIL subsets. **(A)** Global gene expression profile of TIGIT^+^CD8^+^ TIL and TIGIT^–^CD8^+^ TIL subsets. The red dots represent 448 genes with higher expression and the blue dots represent 225 genes with lower expression in TIGIT^+^CD8^+^ TILs compared to TIGIT^–^CD8^+^ TILs. **(B)** Heatmap showing the differentially expressed genes with a fold change greater than 2 involved in chemotaxis, transcription factors and apoptosis. **(C)** Heatmap showing the differentially expressed genes with a fold change greater than 2 involved in the immune response, cytokines and signal transducers. **(D)** Overrepresentation of the functional pathway network. Gene Ontology category enrichment was assessed with the BiNGO 3.0.5 plug-in to Cytoscape 3.10.0. The node size indicates the number of genes involved in functional pathways, with upregulated genes depicted in red and downregulated genes depicted in blue to indicate their targeted processes.


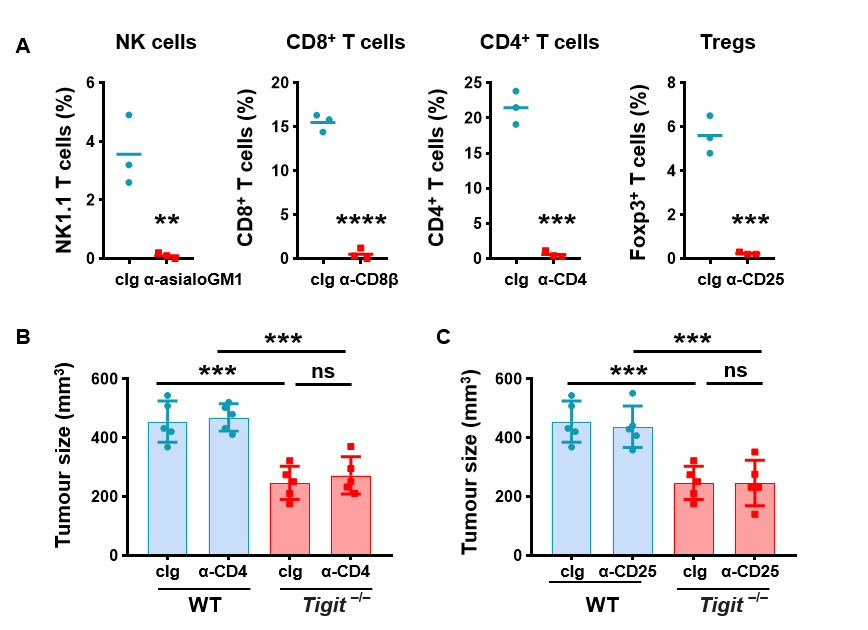


**Supplementary Figure 6** Depletion efficacy of antibodies and tumour size after CD4^+^ T cells or Tregs depletion. **(A)** Mice were treated with anti-asialoGM1, anti-CD8β, anti-CD4, anti-CD25 or their cIg. The proportions of peripheral NK cells, CD8^+^ T cells, CD4^+^ T cells, and Tregs detected on Day 3 by flow cytometry are shown. **(B, C)** *Tigit*^−/−^ and C57BL/6 WT mice were inoculated s.c. with 1 × 10^6^ LLC1 cells and injected i.p. with anti-CD4 **(B)**, anti-CD25 **(C)**, or their cIg. Tumour size on Day 25 after challenge is shown. Results are presented as the means (A) and means ± SD (B, C) and data shown are representative of three independent experiments. ***P* < .01, ****P* < .001, *****P* < .0001, ns, no significant difference; two-tailed Student’s *t*-test (A) and one-way ANOVA with Tukey’s post hoc test (B, C) were used. CIg: control immunoglobulin; SD, standard deviation.


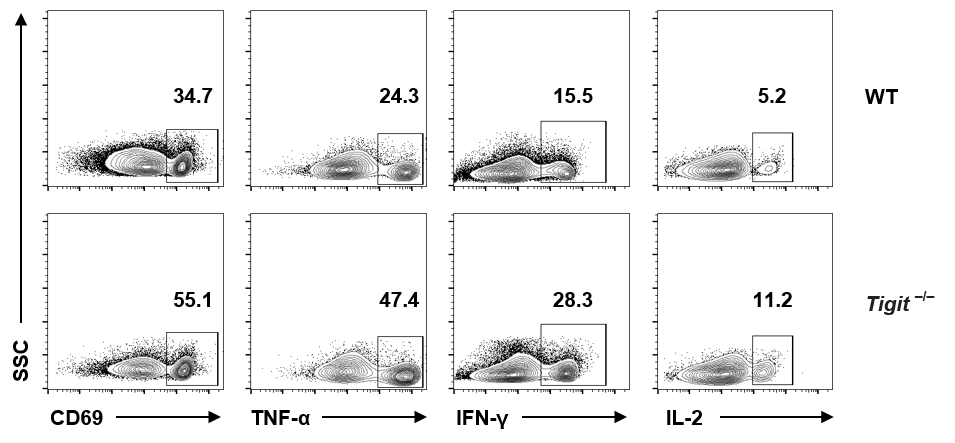


**Supplementary** **Figure 7** Representative flow cytometry plots showing the percentages of CD69, TNF-α, IFN-γ, and IL-2 within the CD8^+^ TIL population in *Tigit*^−/−^ mice and WT mice.


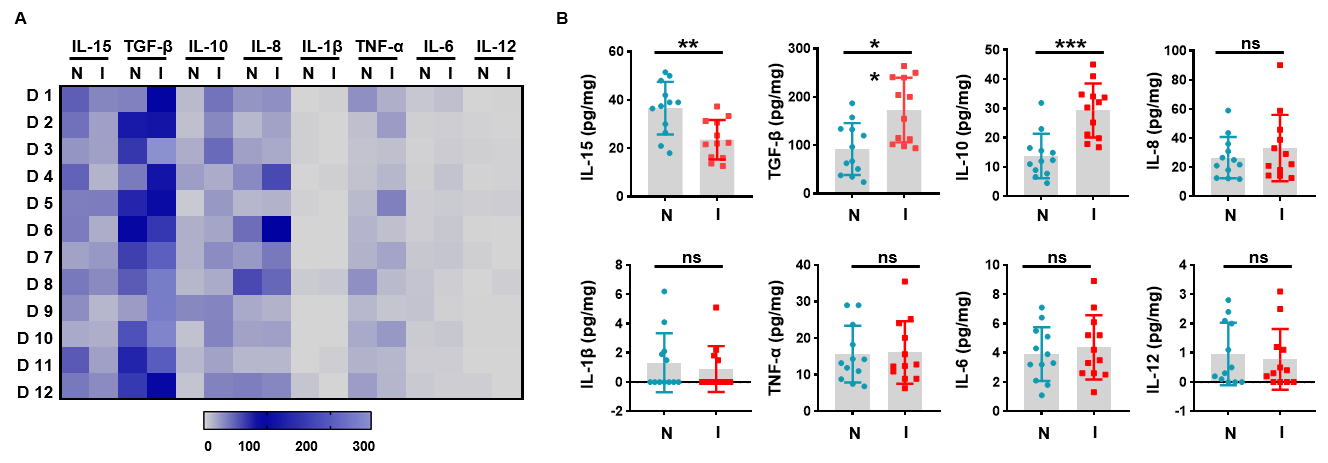


**Supplementary Figure 8** Tissue cytokine levels in LUAD patients. **(A)** Heatmap indicating the expression levels of IL-15, TGF-β, IL-10, IL-8, IL-1β, IL6, and IL-12 in tissue homogenates of normal lung and intratumoural lung (*N* = 12) tissues detected by ELISA. **(B)** Comparison of tissue cytokine levels between normal lung and intratumoural lung specimens. Results are presented as the means ± SD. ***P* < .01, ****P* < .001, ns, no significant difference; two-tailed Student’s *t*-test (B) was used. N, normal lung tissue; I, intratumoural tissue; SD, standard deviation.


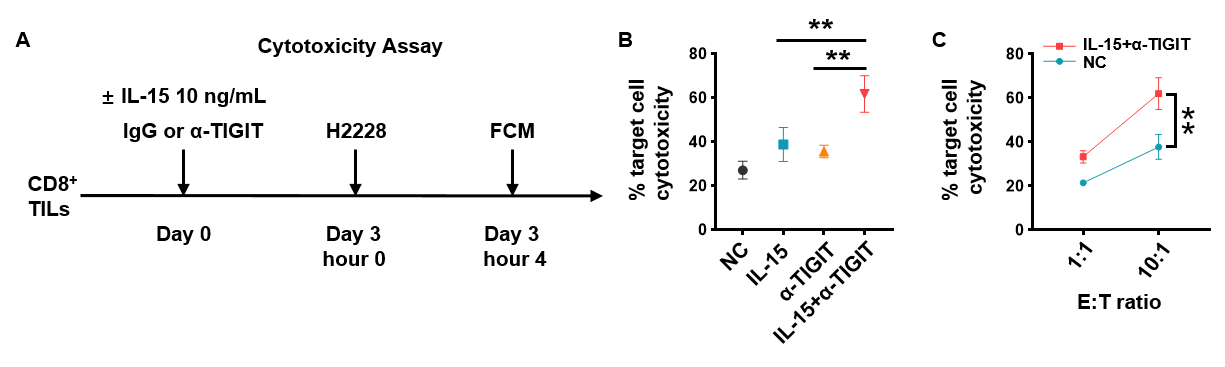


**Supplementary Figure 9** The combination of IL-15 with TIGIT blockade enhances CD8^+^ TIL-mediated cytotoxicity against H2228 cells. **(A)** Flow chart for cytotoxicity assay mediated by CD8^+^ TILs. **(B)** Cytotoxicity percentage of H2228 cells mediated by CD8^+^ TILs following treatment with IL-15 and anti-TIGIT (red), IL-15 alone (green), anti-TIGIT alone (orange) or negative control (dark). **(C)** Cytotoxicity percentage of H2228 cells mediated by CD8^+^ TIL following treatment with IL-15 and anti-TIGIT (red) or negative control (green) at a 1:1 and 10:1 E:T ratio. Results are presented as the means ± SD of at least 3 independent individuals. ***P* < .01; one-way ANOVA with Tukey’s post hoc test (B) and two-way ANOVA (C) were used. FCM, Flow Cytometry; NC, negative control; TIL, tumour infiltrating lymphocyte; SD, standard deviation.


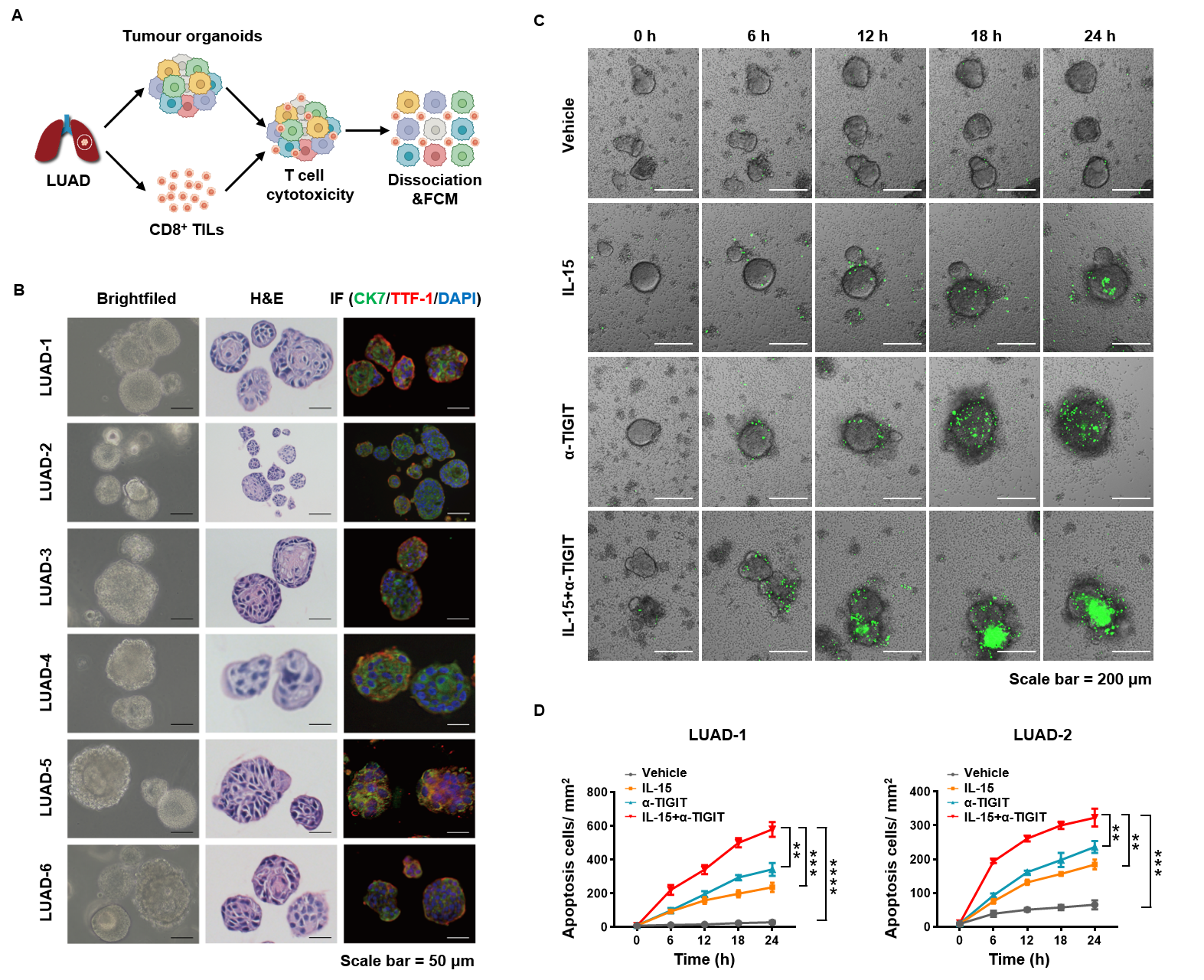


**Supplementary Figure 10** The combination of IL-15 with TIGIT blockade enhances the cytotoxicity against PDOs. **(A)** Schematic depiction of the coculture of PDOs and autologous CD8^+^ TILs. **(B)** Brightfiled microscope, H&E staining and immunofluorescent staining of the LUAD marker CK7 (green) and TTF-1 (red) for organoids. **(C)** The cytotoxicity of autologous CD8^+^ T cells on the PDOs treated with control, IL-15, anti-TIGIT, or their combination. Caspase 3/7 labeled dead cells are shown in green. **(D)** Quantitative statistics of apoptosis cells in **(C)**. Apoptotic cells were computed using Imaris software based on the size and intensity threshold. Results are presented as the means ± SD with error bars representing SD of three view fields. ***P* < .01, ***P* < .001, *****P* < .0001; two-way ANOVA with Tukey’s post hoc test was used. LUAD, lung adenocarcinoma; FCM, flow cytometry; IF, immunofluorescence; SD, standard deviation.

**Supplementary Table 1** Univariate analysis of OS among patients with LUAD according to clinical or immune parameters

|  | OS | | |
| --- | --- | --- | --- |
| Parameter | HR | 95% CI | *P** |
| Clinical parameters |  |  |  |
| Gender (Male) | 1.273 | 1.002 to 1.617 | 0.048† |
| Age (y) | 1.041 | 1.029 to 1.054 | ＜0.001† |
| Metastasis (Y/N) | 4.135 | 3.211 to 5.324 | ＜0.001† |
| Differentiation |  |  |  |
| High | (reference) |  |  |
| Moderate | 1.201 | 0.785 to 1.837 | 0.02† |
| Low | 1.756 | 1.094 to 2.816 | 0.399 |
| Diameter of Tumour (≤5cm) | (reference) |  |  |
| Diameter of Tumour (>5cm) | 0.793 | 0.374 to 1.681 | 0.546 |
| TNM stage |  |  |  |
| I | (reference) |  |  |
| II | 2.300 | 1.595 to 3.317 | ＜0.001† |
| III | 6.844 | 4.940 to 9.483 | ＜0.001† |
| IV | 23.993 | 15.433 to 37.301 | ＜0.001† |
| Immune parameters |  |  |  |
| TIGIT (Low) | (reference) |  |  |
| TIGIT (High) | 1.552 | 1.220 to 1.975 | 0.002† |
| CD96 (Low) | (reference) |  |  |
| CD96 (High) | 1.794 | 0.7464 to 4.311 | 0.077 |
| CD226 (Low) | (reference) |  |  |
| CD226 (High) | 0.959 | 0.809 to 1.041 | 0.096 |

NOTE: All categorical covariates were transformed into numeric codes before they were entered into the Cox model. The numeric codes are as follows: Gender: female = 0, male = 1. Metastasis: N = 1, Y = 2. Pathology Grading: High = 1, Moderate = 2, Low = 3. Tumour diameter: less than 5 cm = 1, more than 5 cm = 2. TNM stage: stage I = 1, stage II = 2, stage III = 3, stage IV = 4. TIGIT: Low = 1, High = 2; CD96: Low = 1, High = 2; CD226: Low = 1, High = 2. *Log-rank P value corrected. † Significant.

Abbreviations: OS, overall survival; HR, hazard ratio; CI, confidence interval.

**Supplementary Table 2** Multivariate Cox proportional hazard analysis for survival time among patients with LUAD

|  | OS | | |
| --- | --- | --- | --- |
| Variable | **HR** | **95% CI** | ***P* *** |
| Before backward selection |  |  |  |
| Gender (Male) | 1.216 | 0.946 to 1.564 | 0.127 |
| Age (y) | 1.058 | 1.044 to 1.071 | ＜0.001† |
| Metastasis (Y/N) | 1.543 | 1.059 to 2.248 | 0.024† |
| TNM stage |  |  |  |
| I | (reference) |  |  |
| II | 2.352 | 1.503 to 3.681 | ＜0.001† |
| III | 5.696 | 3.652 to 8.885 | ＜0.001† |
| IV | 29.619 | 16.819 to 52.161 | ＜0.001† |
| TIGIT | 1.225 | 0.942 to 1.593 | 0.130 |
| CD96 | 2.903 | 1.454 to 5.797 | 0.003† |
| CD226 | 0.888 | 0.693 to 1.586 | 0.351 |
| After backward selection |  |  |  |
| Age (y) | 1.058 | 1.044 to 1.071 | ＜0.001† |
| Metastasis (Y/N) | 1.594 | 1.091 to 2.327 | 0.016† |
| TNM stage |  |  |  |
| I | (reference) |  |  |
| II | 2.198 | 1.414 to 3.419 | ＜0.001† |
| III | 5.466 | 3.508 to 8.518 | ＜0.001† |
| IV | 29.995 | 17.042 to 52.793 | ＜0.001† |
| CD96 | 2.927 | 1.486 to 5.765 | 0.002† |

NOTE: All categorical covariates were transformed into numeric codes before they entered into the Cox model. Numeric codes are as follows: Gender: female = 0, male = 1. Metastasis: N = 1, Y = 2. TNM stage: stage I = 1, stage II = 2, stage III = 3, stage IV = 4. TIGIT: Low = 1, High = 2; CD96: Low = 1, High = 2; CD226: Low = 1, High = 2 *Log-rank P value corrected. †Significant.

Abbreviations: HR, hazard ratio; CI, confidence interval.

**Supplementary Table 3** Clinicopathological characteristics of all patients

| Number of patients | 441 |
| --- | --- |
| Gender (male) | 234(53.1%) |
| Age (years) | 58.4±10.7 |
| OS (days) | 46.6±28.3 |
| Diameter of Tumour (cm) | 2.4±1.4 |
| Metastasis negative | 270(61.2%) |
| Recurrence (+) | 24(5.4%) |
| Differentiation |  |
| High | 41(9.3%) |
| Moderate | 309(70.1%) |
| Low | 91(20.6%) |
| TNM stage |  |
| I | 263(59.6%) |
| II | 58(13.2%) |
| III | 82(18.6%) |
| IV | 38(8.6%) |

Abbreviations: OS, overall survival.
